# The total mass, copy number, and distribution of hormones in the human bloodstream

**DOI:** 10.1371/journal.pbio.3003864

**Published:** 2026-06-22

**Authors:** Ron Sender, Tal Kedar, Yoav Navon, Moriya Raz, Shirley Bikel, Rina Hemi, Ron Milo, Shai Fuchs

**Affiliations:** 1 Department of Plant and Environmental Sciences, Weizmann Institute of Science, Rehovot, Israel; 2 Institute of Pediatric Endocrinology and Diabetes, Edmond and Lily Safra Children's Hospital, Sheba Medical Center, Israel affiliated with Gray Faculty of Medical and Health Sciences, Tel-Aviv University, Tel-Aviv, Israel; 3 Department of Molecular Cell Biology, Weizmann Institute of Science, Rehovot, Israel; 4 Division of Endocrinology, Diabetes and Metabolism, Sheba Medical Center, Ramat Gan, Israel; Stanford University, UNITED STATES OF AMERICA

## Abstract

The human endocrine system orchestrates critical physiological processes, yet a systematic quantitative synthesis of clinically relevant circulating hormones has been lacking. Here, we present a comprehensive, integrative analysis of circulating human hormones, leveraging clinically validated reference intervals across major endocrine subsystems. We use clinically validated reference intervals that we further validate using published datasets. Our analysis reveals that the total mass of circulating hormones is approximately 40 ± 2 mg. We find that this mass in healthy young adults is dominated by Adiponectin and DHEAS, which constitute over 90% of both total hormone weight and copy number. We show there are on the order of a million hormone molecules per cell in the human body. Females have about half the number of circulating hormone molecules compared to males. Across 56 hormones with curated affinity data, free (receptor-available) concentration correlates with receptor binding affinity, with class-specific scaling. Bioavailability mechanisms segregate by chemical class, consistent with chemical structure constraining available buffering strategies. Together, these data provide a quantitative reference for the human endocrine system and highlight relationships linking receptor affinity, bioavailability, and chemical class.

## Abstract 2

The human endocrine system orchestrates critical physiological processes, yet a systematic quantification of circulating hormones has been lacking. This study provides an integrative analysis of all known circulating human hormones, revealing patterns in their abundance and distribution.

## Introduction

The endocrine system regulates energy balance and distributes nutritional energy across various levels of physiological activity and homeostasis. It governs critical functions including growth, metabolism, reproduction, and stress response through a network of circulating hormones [1, 2].

While comparing hormone concentrations within specific endocrine circuits (e.g., TSH and free T4) is common in clinical diagnostics and research, and cross-species comparisons of one or a few hormones have proven valuable in evolutionary studies [3, 4], a comprehensive, integrative analysis of circulating hormones has been lacking. Though several databases catalog messenger molecules [5–7], a quantitative comparison across hormones has not been performed. Here, we present a comprehensive, integrative, quantitative analysis of human hormone abundances, focusing on clinically assayed hormones with established reference intervals, assessing their relative abundances within a unified framework.

Clinical reference ranges, first introduced as a concept in the 1960s [8, 9], represent an ongoing effort to acquire and analyze data from healthy subjects according to standardized guidelines [10–12]. As such, they provide insight into the physiology of human blood analytes. Typically, the reference interval encompasses the central 95% of a reference population, centered on the median [12] with some exceptions derived from physiological endpoints (e.g., fasting glucose) [13].

Using established clinical reference intervals, we compiled a database of 63 hormones, mostly routinely assayed in medical laboratories (with a minority primarily measured in research and specialty contexts), spanning the endocrine glands and hormone-secreting tissues. We then performed an integrated analysis of abundance patterns, validated our findings through examination of published cohorts, and extended our analysis to available data for the wider human hormonal repertoire.

## Results

### Two hormones comprise ≈90% of the circulating mass

**Fig 1:**
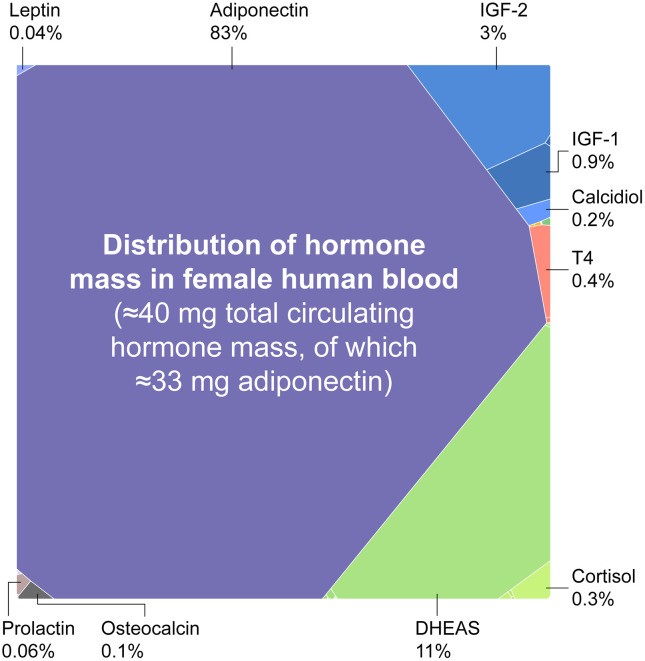
The distribution of hormone mass in human blood is dominated by two hormones. Voronoi diagrams illustrating the distribution of the circulating hormones by mass in women (see Fig A in S1 Appendix for men). The area of each polygon is proportional to the relative mass of each hormone. Colors represent different endocrine systems or hormone types. The top 10 contributors are labeled with their names and percentage contributions. Adiponectin and DHEAS dominate the hormone mass in both sexes, together accounting for approximately 93% of the total. Total circulating hormone mass is approximately 40 mg in both women and men. Underlying data: S1 Data, sheet Hormone_abundance. Generated using the Proteomaps tool (http://bionic-vis.biologie.uni-greifswald.de/).

The total mass of hormones circulating in the blood of a healthy young adult is approximately 40 mg (≈41.5 mg in men and ≈39.5 mg in women). This mass is dominated by two hormones: adiponectin and dehydroepiandrosterone sulfate (DHEAS). Adiponectin, primarily produced by adipose tissue, contributes roughly 74% of total mass in men and 83% in women (≈31 mg in men, ≈33 mg in women). DHEAS, a sulfated reservoir form of an androgen precursor synthesized in the adrenal glands, differs between sexes: it comprises ≈20% of the total in men (≈8 mg) but only 11% in women (≈4 mg) (Fig 1, Fig A in S1 Appendix).

Following these two dominant hormones, the liver-derived insulin-like growth factors, IGF-2 and IGF-1, are the next most abundant in both sexes. The remaining 59 clinically assayed hormones each contribute less than 0.5% to the total circulating hormone mass, collectively representing only 3%.

The significant contributions of adipose tissue (through adiponectin) and liver (through IGFs) to the total circulating hormone mass highlight the important role of non-classical endocrine tissues in overall endocrine function (Fig B in S1 Appendix).

While total mass provides insight into biosynthetic investment, the biological impact of hormones depends on their molecular interactions with receptors. Therefore, we next examined the number of circulating molecules for each hormone to understand their potential signaling capacity.

### Hormone molecule copy numbers show dominance of the adrenal cortex and sexual dimorphism

**Fig 2:**
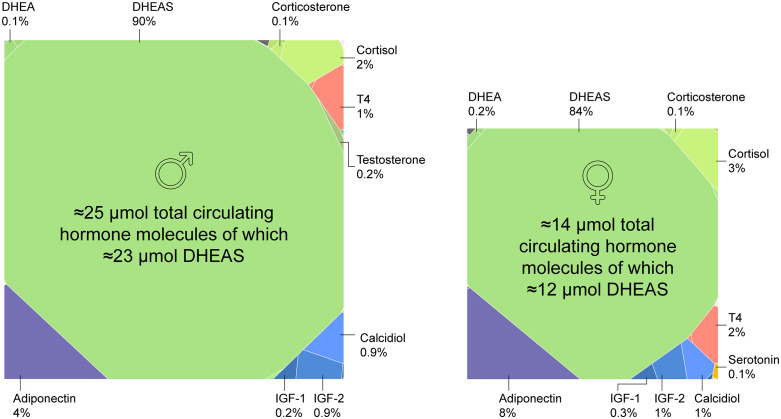
The distribution of hormone molecule copy numbers in males and females. The distribution is represented by a Voronoi diagram, such that the area of each polygon is proportional to the fractional abundance of a given hormone. Juxtaposing male (left) and female (right), the area of the square is proportional to the total number of circulating molecules. In males, the total count of hormone molecules (≈25 μmol or ≈1.5 × 10^19^ molecules) is nearly double that in females (≈14 μmol or ≈8 × 10^18^ molecules). Underlying data: S1 Data, sheet Hormone_abundance. Generated using the Proteomaps tool (http://bionic-vis.biologie.uni-greifswald.de/).

Next, we evaluated the number of circulating molecules per hormone, which reflects the potential for receptor-mediated signaling. In a reference male, we estimate approximately ≈25 µmol in total, compared to 14 µmol in women. This is equivalent to ≈15 × 10^18^ and ≈8 × 10^18^ circulating hormone molecules in men and women respectively, showing that the female hormone molecule copy number is nearly half that of male (Fig 2 and Fig C in S1 Appendix). Given that the estimated number of cells in the male and female body is ≈3 × 10^13^ and ≈2 × 10^13^, respectively [14], we find that at any given moment, there are close to a million circulating hormone molecules per cell in the human body.

DHEAS, a sulfated isoform of the adrenal androgen precursor, dominates circulating hormones, accounting for 90% and 83% of total hormone molecules in males and females, respectively (Fig 2). Collectively, adrenocortical steroid hormones constitute approximately 90% of circulating hormone molecules in both sexes. Adiponectin shows sexual dimorphism: it comprises 8% of circulating hormone molecules in females, double its 4% contribution in males. Adiponectin’s molecular count contrasts with its overall dominance in total hormone mass, reflecting its high molecular weight.

### Circulating hormone abundance spans eight orders of magnitude

**Fig 3:**
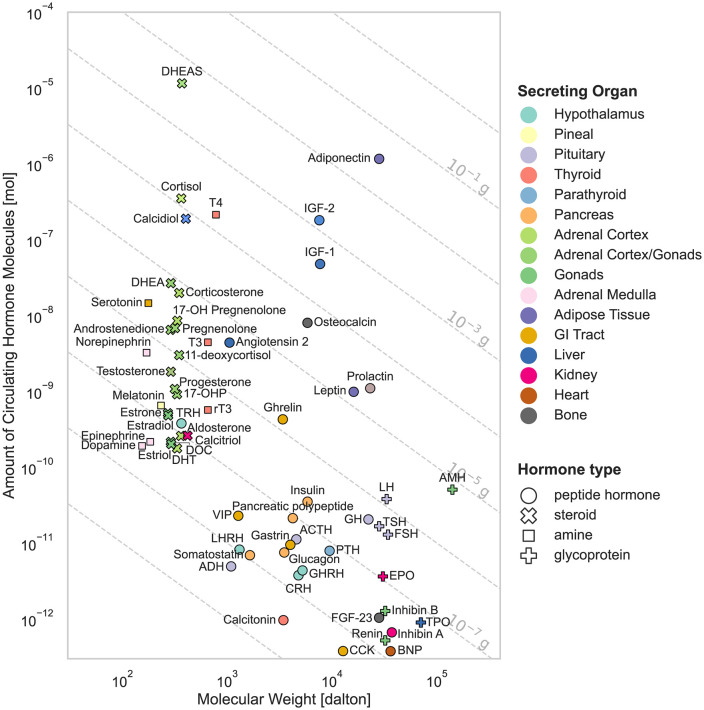
Distribution of circulating hormones by molecular weight and abundance in females. Hormones are plotted by molecular weight (x-axis) and circulating abundance (y-axis). Colors represent secreting glands/tissues, while shapes indicate hormone types (e.g., peptide, steroid). Diagonal iso-mass lines show total circulating mass in grams (product of molecular weight and abundance). For gonadotropins and sex hormones, median copy numbers are based on reference intervals from the phase of the menstrual cycle when levels are lowest per hormone. Corresponding distribution for males is presented in Fig E in S1 Appendix. Underlying data: S1 Data, sheet Hormone_abundance. Code: Zenodo DOI https://doi.org/10.5281/zenodo.20110628.

We examined the distribution of hormone abundances across molecular weights and hormone types. Fig 3 illustrates the relationship between hormone molecular weight and circulating molecule copy numbers. Hormone molecular weights span three orders of magnitude and cluster strongly by hormone type: amine hormones and steroids (10^2^–10^3^ daltons), followed by peptide hormones (10^3^–10^4^ Da), and then glycoproteins (10^4^–10^5^ Da) (Fig D in S1 Appendix).

Circulating hormone molecule counts span a hundred million-fold range, and do not manifest any clear pattern. Because total circulating mass per hormone is the product of molecular weight and molar abundance, diagonal iso-mass lines in Fig 3 reveal hormones with similar total circulating masses. Classification by molecular weight and tissue origin does not, on its own, explain hormone abundance. To identify determinants of hormone abundance, we examined receptor binding affinity, bioavailability, and elimination half-life.

### Circulating hormone abundance partially scales with receptor binding affinity in a hormone class-dependent manner

To investigate potential determinants of the widely varying hormone circulating abundance, we assembled binding affinity data for hormone-receptor pairs and examined whether concentration relates to receptor binding affinity, and whether incorporating free fraction, half-life, or hormone class improves explanatory power. We mined public databases and primary literature to compile equilibrium dissociation constants (Kd) for 56 hormones and their primary cognate receptor, prioritizing measurements performed under physiological conditions when available (S2 Data, sheet ‘Kd’). In parallel, we curated two additional datasets: receptor-available fractions (accounting for protein binding and, where applicable, reservoir/activation steps) and circulating half-lives (S2 Data, sheets ‘Bioavailability’ and ‘Half_life’).

Linear regression in log–log space revealed a significant relationship between circulating hormone concentration and receptor Kd (*R*^2^ = 0.29, *p* < 0.001) (Fig H in S1 Appendix). Because receptor engagement depends on the free (unbound) ligand concentration, we repeated the analysis using estimated free concentrations; model performance increased slightly (*R*^2^ = 0.31), and we retained free concentrations for downstream analyses as a physiologically interpretable estimate of the ligand pool available for receptor interaction. Adding circulating half-life as a covariate did not improve model performance (adjusted *R*^2^: 0.293 → 0.264; AIC: 162.1 → 168.5), indicating no incremental explanatory power from half-life in this linear framework.

**Fig 4:**
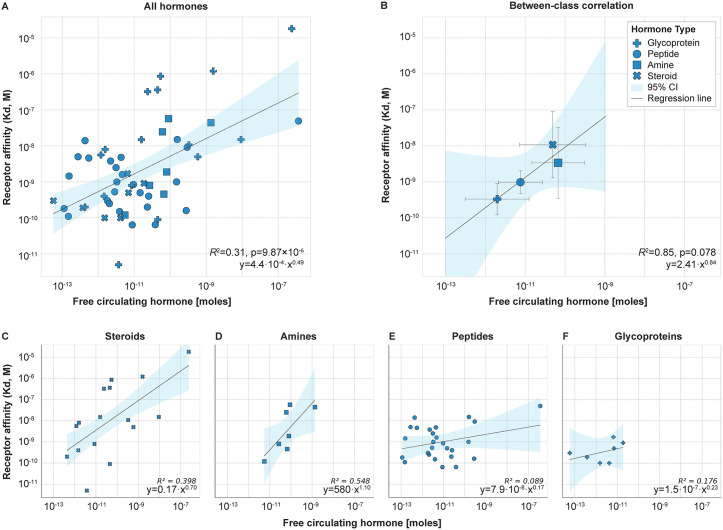
Receptor affinity scales with circulating hormone availability, with class-dependent scaling. **(A)** Log–log relationship between receptor binding affinity (Kd, M) and free (receptor-accessible) circulating hormone count [moles] across hormone–receptor pairs (linear regression fit ± 95% CI; *R*^2^ = 0.306, *p* = 9.9 × 10^−6^). **(B)** Between-class (“ecological”) relationship using class means (*n* = 4 classes; shown as an exploratory summary; *R*^2^ = 0.85, *p* = 0.078). **(C–F)** Within-class log–log relationships for steroids **(C)**, amines **(D)**, peptides **(E)**, and glycoproteins **(F)**, with linear regression fits and 95% confidence bands. The class-stratified model (*R*^2^ = 0.46, *F*(7, 48) = 5.89, *p* = 5.7 × 10^−5^) significantly improves fit over the single-slope model, supporting class-dependent scaling. Individual class slopes differ numerically but not significantly from the reference class (amines): steroids *p* = 0.45, peptides *p* = 0.08, glycoproteins *p* = 0.19. Underlying data: S2 Data, sheets Master_table and Kd. Code: Zenodo DOI https://doi.org/10.5281/zenodo.20110628.

Allowing both intercepts and slopes to vary by hormone class (amines, peptides, glycoproteins, steroids) significantly improved model fit (Δ*R*^2^ = 0.16, *F*(7,48) = 5.89, *p* = 5.7 × 10^−5^), indicating that concentration–affinity scaling is structured by the hormones’ chemical class. The estimated power-law exponents vary across classes, ranging from *β* = 0.17 (peptides) to *β* = 1.10 (amines), with intermediate values for glycoproteins (*β* = 0.52) and steroids (*β* = 0.70) (Fig 4C–4F). Individual classes contain too few hormones to support well-powered tests of within-class slopes. At the level of class means, the four groups occupy distinct positions along the concentration–Kd relationship (*R*^2^ = 0.85, *n* = 4 classes, *p* = 0.078; Fig 4B).

### Mechanisms of sequestration vary by hormone class

**Fig 5:**
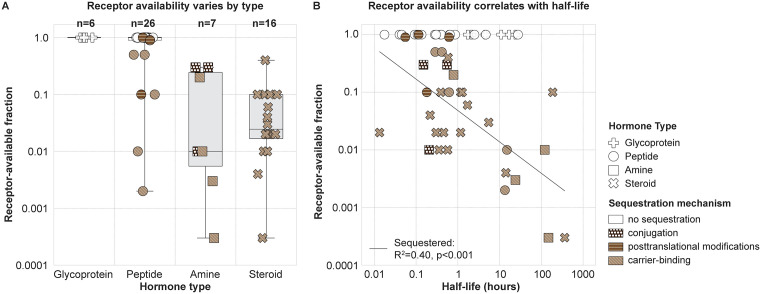
Sequestration and bioavailability vary by hormone type and correlate with half-life. **(A)** Receptor-available fraction by hormone class. Boxes show median and IQR; whiskers extend to 1.5 × IQR; points are individual hormones. Fill patterns denote sequestration mechanism. Receptor availability differs significantly across classes (one-way ANOVA, *F* = 39.68, *p* < 0.001) and associates with sequestration mechanism (*χ*^2^ = 60.31, p < 0.001, Cramér’s *V* = 0.61). **(B)** Receptor-available fraction vs. half-life (log_10_-scaled axes). Among sequestered hormones (filled symbols, *n* = 32), availability decreases with increasing half-life (*R*^2^ = 0.40, slope = −0.54, *p* < 0.001; regression line shown). Non-sequestered hormones (open symbols, *n* = 23) were excluded from the fit. Underlying data: S2 Data, sheets Master_table, Bioavailability, and Half_life. Code: Zenodo DOI https://doi.org/10.5281/zenodo.20110628.

Receptor-available fraction and circulating half-life contributed little additional explanatory power for hormone abundance beyond affinity-based models (Δ*R*^2^ < 0.01), yet their systematic curation revealed strong class-structured organization. We classified four dominant strategies shaping receptor availability: negligible/no sequestration (receptor-available fraction ≈ 1), conjugation, post-translational processing/activation steps, and carrier binding (reversible sequestration, mostly by protein carriers). These strategies segregated by hormone chemical class (*χ*^2^ = 60.31, df = 9, *p* < 0.001; Cramér’s *V* = 0.61; Fig 5A and Fig I in S1 Appendix): glycoproteins showed minimal sequestration (7/7), steroids showed carrier binding (16/16), amines partitioned between conjugation and carrier binding (3/7 and 4/7, respectively), and peptides exhibited greater diversity. Consistent with this mechanistic segregation, receptor-available fraction differed significantly across hormone classes (one-way ANOVA: *F*_3,51_ = 39.68, *p* < 0.001; Fig 5A), with steroids displaying markedly lower receptor-available fractions than glycoproteins and peptides (Tukey HSD post-hoc: *p* < 0.001 for both comparisons). Among sequestered hormones, receptor-available fraction was inversely associated with circulating half-life (*R*^2^ = 0.40, slope = −0.54, *p* < 0.001, *n* = 32; Fig 5B). Together, these analyses suggest a structured relationship in which chemical class constrains feasible sequestration mechanisms, mechanism choice shapes receptor-available fraction, and stronger sequestration is associated with longer circulating residence times.

### Sex-specific differences in hormone abundance

In our analysis, we used reference intervals available for both young adult females and males. The difference between males and females emanates from two factors—serum volume, whereby female serum volume is 2.4 L while male serum volume is 3 L [15]; and differences in median concentrations. For hormones with fluctuating levels, such as those varying throughout the menstrual cycle, we adhered to using the reference values corresponding to the stage where the hormone level is at its minimum. However, even substantial menstrual-related fluctuations, such as a 13-fold increase in LH during ovulation, exert a negligible influence on the total hormone mass and count.

Out of the 63 hormones and hormone isoforms in our cohort, 31 have the same reference intervals in males and females. The main differences, as could be expected, are in sex hormones and adipokines, the latter corresponds to the higher percent body fat mass in females. These differences are most pronounced in the two main contributors to hormone mass and count: adiponectin levels are generally higher in females due to greater body fat percentage, while DHEAS levels are typically higher in males, reflecting its role as an androgen precursor.

### Validation and comprehensive analysis of hormone mass estimation

Our hormone abundance analysis is based on using the geometric mean of reference interval bounds as a proxy for the median in a reference young adult population. To estimate the error in this approach, we examined the published studies reporting hormone levels in healthy young adult cohorts. We focused on adiponectin and DHEAS, which comprise ≈93% of the total hormone mass and copy number.

Analysis of the seven published studies on hormone levels in young adults revealed literature-derived median expressions (Table 1 and Table B in S1 Appendix). From these, we calculated total circulating masses of 26 mg (men) and 33 mg (women) for adiponectin, and 10 mg (men) and 5 mg (women) for DHEAS. The sum of these two hormones is therefore 36 mg in men and 38 mg in women. In comparison, our main analysis yielded 39 mg in men and 37 mg in women for this sum. This indicates an overestimation of up to 11% for men and 3% for women in our approach. Given that all other hormones combined contribute less than 3 mg to the total, further analysis of these minor contributors is unlikely to significantly alter this error estimation.

**Table 1:** Literature-derived estimation of median and distribution of adiponectin and DHEAS concentrations in young adults.

		Median [µg/ml]		Total variation [µg/ml]	
hormone	sex	estimate	CI95%	50%	90%
Adiponectin	F	13.7	13.2–14.2	10.2–18.5	6.6–28.2
Adiponectin	M	8.5	8.1–8.8	6.1–11.6	3.9–18.3
DHEAS	F	1.9	1.8–2.4	1.5–2.9	1.1–4.7
DHEAS	M	3.2	3.0–3.5	2.4–4.2	1.6–5.9

This table presents the literature-derived median estimates and total variation for adiponectin and DHEAS concentrations stratified by sex. Median estimates are accompanied by their 95% confidence intervals (CI95%). Total variation is represented by interquartile ranges covering 50, and the 90% of the population, respectively. The data were obtained from a systematic review of studies involving individuals aged 20–30 years, with bootstrapping methods applied to estimate central tendency and variability. Underlying data: Table C in S1 Appendix.

To quantify the coverage of our primary analysis, we conducted a comprehensive screening of 7,996 candidate entries from UniProt and HORDB databases, and in the seminal textbooks of pediatric and adult clinical endocrinology [5, 6, 16–18]. Our evaluation of an additional 124 endocrine molecules beyond the primary cohort revealed they contribute approximately 2.2 mg to the total circulating mass, representing a minor (~6%) potential underestimation. This multi-tiered validation, comparing our clinical reference-based estimates with published cohorts for high-abundance markers like adiponectin and DHEAS, provides a total hormone mass of 41.5 ± 1.5 mg for men and 39.5 ± 1.5 mg for women. We conclude that our error estimation based on the geometric mean of reference interval boundaries is ≈5% of the total circulating hormone mass in the reference human. This error margin affirms that our main analysis captures the vast majority of circulating hormone abundance.

### Biological variation context

For 25 hormones with available entries in the EFLM Biological Variation Database [19], between-subject variability (CVg) generally exceeded within-subject variability (CVi) (median CVg ≈ 0.32; median CVi ≈ 0.17; Table D in S1 Appendix). Variability did not segregate by biochemical class, with both low- and high-variability hormones present in each class (Table D in S1 Appendix). The lowest variability was observed for thyroid hormones (T3 total: CVg ≈ 0.12, CVi ≈ 0.06), whereas higher variability was observed for prolactin (CVg ≈ 0.64, CVi ≈ 0.45) (Table D in S1 Appendix). Hormones with pronounced circadian or pulsatile dynamics are represented here by reference-interval–anchored summary concentrations rather than time-resolved profiles (see Methods).

## Discussion

We assembled a quantitative overview of circulating hormone abundance in humans by combining clinically defined reference intervals with published datasets and applying a multi-step validation procedure. Using this approach, we estimate that the total blood hormone mass is 41.5 ± 1.5 mg for men and 39.5 ± 1.5 mg for women with an estimation error under 5%. This framework brings hormone families that are typically discussed separately onto a common scale, allowing direct comparisons of their circulating pools in units of both mass and molarity.

Our account highlights an eight orders-of-magnitude spread in circulating hormone molecule count, framing the question of what constraints shape the landscape of hormone abundance. Recent work has suggested that endocrine gland size scales with the size of target tissues [20] raising the possibility that production capacity and tissue mass could influence circulating pool sizes. Consistent with this idea, large-mass tissues such as adipose and liver contribute substantially to the circulating mass of some hormones in our estimates, including adiponectin and IGFs, respectively. However, tissue mass alone is not sufficient to systematically account for the overall abundance patterns across hormones. Moreover, the aggregate mass of circulating hormones (~40 mg) is negligible relative to typical daily whole-body biomass turnover (~100 g) [21], suggesting that global biomass supply is unlikely to be limiting. These considerations motivate asking whether receptor affinity and class-linked buffering mechanisms offer a clearer framework than production capacity alone.

We then examined the relationship between hormone abundance and receptor binding affinity (Kd) across 56 hormones with available binding data. Total circulating concentrations showed a positive correlation with Kd (*R*^2^ = 0.29, Fig H in S1 Appendix): higher-abundance hormones generally bind their cognate receptors with lower affinity. These patterns are compatible with concentration-affinity matching principles analogous to those in metabolic networks [22], where substrate concentrations are maintained high enough to keep enzyme binding sites occupied. Accounting for bioavailability to estimate free (receptor-accessible) concentrations improved the correlation with binding affinity modestly (*R*^2^ = 0.31 versus 0.29, Δ*R*^2^ = 0.02, *p* = 9.9 × 10^−6^, Fig 4A). This modest improvement is consistent with established observations that when tissue uptake is elimination-limited, intracellular hormone concentrations are proportional to free serum concentrations regardless of uptake mechanism [23]; the limited divergence between total and free predictive power reflects moderate binding fractions (10%–50%) for most hormones in our dataset. The remaining variance likely reflects unmeasured factors such as receptor abundance, receptor subtype diversity, cofactor requirements, tissue-specific expression, temporal dynamics, and blood-tissue compartmentalization.

We focused on endocrine ligands with standardized clinical measurement in blood, spanning steroids, amines, peptides, and glycoproteins. Locally acting mediators (eicosanoids, gaseous messengers) and predominantly immune signaling molecules (many cytokines), and growth factors are not included. The included classes differ in size, solubility, carrier binding, and clearance, properties that are expected to influence both circulating pool size and receptor-accessible exposure [17, 18].

We next asked whether the concentration–affinity relationship follows a single rule across hormones or instead differs by chemical class. The class separation in Fig 4 is consistent with class-linked constraints, such as size, solubility, carrier binding, and clearance, which affect both circulating pool size and receptor-accessible exposure. This interpretation is reinforced by bioavailability patterns (Fig 5 and Fig I in S1 Appendix): sequestration mechanisms segregate sharply by class, producing systematically different free fractions, and among sequestered hormones stronger buffering is associated with longer circulating residence times (Fig 5B). Together, these findings suggest that chemical class is an organizing axis because it constrains the buffering and clearance strategies available to a hormone, which in turn shapes receptor-accessible exposure.

To examine whether high-abundance hormones share common features, we defined a “top set” as the union of the top five hormones ranked by total mass and by molarity, evaluated separately in each sex, which yields a stable set of seven hormones that recurs across both measures and sexes (with rank order varying). These seven span an array of physiological domains: energy metabolism and insulin sensitization (adiponectin), growth and tissue maintenance (IGF-1, IGF-2), the stress response (cortisol), androgen precursor biology (DHEAS), thyroid hormone regulation (T4), and calcium and bone homeostasis (calcidiol), yet they share common circulation-level features. Six of the seven are extensively carrier-protein bound; three of these, T4, calcidiol, and DHEAS, are additionally upstream precursors requiring enzymatic activation to more potent forms. T4 and calcidiol engage the same receptors as their active products but with 10- to 30-fold and ~1,000-fold lower affinity, respectively [24–26]. DHEAS is not an androgen-receptor ligand, but has been reported as a candidate sigma-1 agonist in pharmacological assays; accordingly, we map DHEAS to sigma-1 as an operational assignment [27, 28], and validate through sensitivity analysis (methods). Last, adiponectin employs a distinct buffering mechanism: its insulin-sensitizing and anti-inflammatory activity depends on assembly into high-molecular-weight oligomers rather than release from carrier protein [29]. Taken together, these seven illustrate a common theme: large circulating pools are usually maintained by mechanisms that decouple total abundance from immediate receptor exposure. Adiponectin is a case in point: total mass is the largest of any circulating hormone, yet it barely figures in routine clinical practice. Conversely, low-abundance hormones such as insulin and GnRH exert outsized physiological effects through high receptor affinity and signal amplification. Abundance therefore reflects circulation-level buffering architecture, not physiological importance.

While total hormone mass is similar between sexes, we observed important sex-specific differences in hormone count. Men have nearly double the number of circulating hormone molecules compared to women, primarily due to variations in DHEAS levels. The X-linked Steroid sulfatase enzyme, which negatively controls DHEAS abundance, escapes X-inactivation, leading to 2-fold higher expression and activity in females [30, 31], contributing to these differences. While menstrual cycle stages minimally impact overall hormone mass due to the relatively low abundance of gonadotropins and estrogen, pregnancy profoundly alters the female hormonal landscape, with human placental lactogen alone reaching over 20 mg in the third trimester (50% of total hormone mass in non-pregnant women), attesting to the placenta as a high-biomass hormonally active organ [32, 33].

This study quantifies clinically assayed circulating hormones in adults, rather than attempting an exhaustive census of endocrine signaling molecules. To contextualize how representative this quantified subset is, we assembled a broader catalogue of endogenous hormones using a conventional endocrinology scope and then asked what fraction is covered by standardized clinical measurement. Under this definition, the quantified subset comprises roughly one-third of catalogued hormones yet captures ~95% of total circulating hormone mass, providing a substantial sample for evaluating whether abundance exhibits reproducible structure. Extending the same quantitative framework to the remaining catalogue is an important next step, but will likely be noisier in the near term, since many non-clinically assayed hormones are less well characterized with respect to circulating ranges, binding, and clearance and are supported by a thinner literature base.

Several limitations of our analysis should be noted. Reliance on reference intervals from a primary resource necessarily neglects variance across laboratories, assay platforms, and measurement matrices (serum versus plasma; immunoassay versus mass spectrometry). Moreover, the notion of a single “reference healthy human” elides systematic population differences. For example, adiponectin levels have been reported to be 30%−40% lower in self-reported African-Americans compared to self-reported White or Hispanic participants [34, 35], underscoring the need for population-specific analyses. Within individuals, our approach also sets aside the temporal pattern of many hormones (circadian rhythms, pulsatility, seasonality, and state dependence). These sources of heterogeneity are likely to contribute primarily to residual scatter in cross-hormone comparisons; however, because the log–log relationships analyzed here span multiple orders of magnitude, moderate shifts in any single reference interval are unlikely to account for the qualitative scaling patterns we report.

Placing diverse hormone families on a common quantitative scale reveals reproducible structure. Circulating concentration correlates modestly with receptor affinity, scaling behavior differs across chemical classes, and bioavailability mechanisms segregate by class. These patterns are consistent with concentration–affinity matching ideas, with bioavailability providing a tunable layer shaped by constraints on solubility, clearance, and receptor access. The relationships remain correlational and explain only part of the variance, but they provide a quantitative baseline that highlights regularities and outliers and motivates testable hypotheses about endocrine organization.

Future work can link these abundance patterns to receptor abundance and tissue distribution, carrier binding stoichiometry, and time-resolved secretion and clearance. Broadening coverage to the full annotated hormone list, across the pediatric and aging human, special physiological states such as pregnancy and the postprandial period, and pathologies from obesity to infectious disease, would provide stronger tests of generality and refine the inferred scaling relationships.

## Methods

### Data sources and reference values

We obtained clinical reference values primarily from the Mayo Clinic’s public test catalog, which compiles ranges used in routine clinical practice and cites Tietz Fundamentals of Clinical Chemistry and Molecular Diagnostics as a key reference [13]. When Mayo did not provide a reference interval, or when additional context was needed (e.g., manufacturer insert, platform-specific interval, or population qualifier), we annotated alternative sources alongside the extracted interval (Table A in S1 Appendix).

### Reference human model and reference conditions

All calculations use a reference young–adult model. Unless otherwise noted, the reference male and female were assumed to be 73 and 60 kg, respectively [15]. Where an age parameter was required, we used 25 years as a modeling assumption. Concentrations were interpreted as serum values measured under standard clinical sampling conditions (healthy, non-pregnant, and typically morning/fasting). For hormones with cyclic physiology (e.g., gonadotropins and ovarian steroids), we used the phase of the menstrual cycle associated with the lowest values, consistent with the aim of estimating baseline circulating pools. Serum volumes used to convert concentrations to circulating pools were 3.0 L for males and 2.4 L for females, based on prior whole-body blood and serum volume estimates [15].

### Deriving representative concentrations from reference intervals

Representative concentrations were derived from reference intervals by taking the geometric mean of the lower and upper bounds. This choice reflects the approximate lognormal distribution of many laboratory analytes, including hormones [8, 36]. When the lower bound of a reference interval was reported as 0, we treated the value as left-censored and substituted half the assay limit of detection (LOD/2) as the lower bound, a standard convention for lognormal censored data [37]. LOD values were compiled from assay documentation and are provided in S1 Data (sheet: LOD).

### Calculation of total circulating mass, molarity, and molecule counts

Reference concentrations were converted to mass concentration (pg/ml) and multiplied by sex-specific serum volume to obtain the total circulating mass per hormone. Molar abundance was obtained by dividing mass by molecular weight and converting to molar units; and circulating molecule counts were obtained by multiplying moles by Avogadro’s number. For hormones that circulate bound to carrier proteins, we used the molecular weight of the hormone itself (not the protein–hormone complex) for molar conversions to maintain comparability across mechanisms of bioavailability. The vast majority of clinical hormone assays use serum as the measurement matrix; a small number of hormones (ACTH, PTH, and renin in some assays) are preferentially measured in plasma due to specific stability requirements, and we used the matrix specified in the clinical reference source for each hormone.

### Unit conversions

For hormones reported in activity concentration units (IU/L, mIU/L), we converted to mass concentration units using ratios derived from National Institute for Biological Standards and Control (NIBSC) reagent data sheets (S1 Data, sheet: AU_conversion). Molecular weights were retrieved from the PubChem database. For protein-bound hormones, we used the weight of the unbound molecule. For hormones assayed as subunits or complexes (e.g., glycoprotein hormones), we used the molecular weight of the intact circulating hormone for molar conversions.

### Hormone–receptor binding affinity (Kd) curation

Receptor binding affinities (Kd, Ki, or IC50) were curated for each hormone’s known receptor(s) through two independent searches. The IUPHAR/BPS Guide to Pharmacology database (GtoPDB; accessed 10 June 2025) was queried for endogenous hormone–receptor interactions and associated affinity values. An independent agentic literature search was performed using GPT-4 (OpenAI) to retrieve candidate affinity data and supporting PubMed identifiers for each hormone. All candidate entries from both sources were manually reviewed: each retrieved PMID was individually accessed and the reported affinity value, assay type, and molecular context were verified against the original publication. Only values confirmed in primary sources were included in S2 Data.

The affinity table in S2 Data records all known receptors for each hormone but assigns one as the primary receptor used in downstream calculations. Assignment was based on canonical target identity (e.g., cortisol and the glucocorticoid receptor) or, when no single canonical target was evident, on highest receptor abundance in the canonical target tissue. Where neither criterion was sufficient, selection rationale was specified in the comments field. Primary receptor assignments were reviewed by the corresponding author (S.F.) and locked prior to statistical analysis (author R.S).

Values were recorded as equilibrium dissociation constants (Kd) when available. When Kd was not reported, we captured the closest equilibrium binding proxy provided by the source (e.g., Ki from competition binding) and recorded the affinity type explicitly. Reported affinities were converted to molar units. For each hormone–receptor pair, we preferentially selected measurements on human receptors (native or recombinant) under near-physiological conditions. When multiple values were available, we favored full-length receptor assays closest to the relevant biological context. If human data were unavailable, we accepted measurements from closely related mammalian species, with species and assay context documented in S2 Data. In cases where Ki values were derived from competition assays, we retained the reported Ki and, when the source provided sufficient information, documented the conversion assumptions used (e.g., radioligand concentration and reference Kd). When only IC50 values were available, Kd was estimated using the Cheng–Prusoff equation where the source provided the necessary assay parameters (radioligand concentration and its Kd); the derivation basis was recorded in S2 Data.

### Bioavailability and receptor-available fraction

Bioavailable fractions and sequestration mechanisms were curated through AI-assisted literature searches (GPT-4.5, OpenAI) followed by individual manual verification of each source against the original publication. For each hormone we recorded: (i) the molecular species quantified by routine clinical assays (total hormone or free hormone), (ii) the circulating molecular form considered receptor-available, and (iii) an estimated fraction linking the clinically assayed pool to the receptor-available pool. Known sequestration mechanisms were recorded, including plasma carrier binding, reversible conjugation, and posttranslational modifications; hormones with no significant binding were annotated as “no sequestration.” When the clinical assay measures the receptor-available species directly (e.g., free T3), the bioavailable fraction for that analyte was set to 1.0; a separate column records the free fraction relative to total circulating hormone (e.g., the percent of total T3 that is free) to allow conversion between reporting conventions. Bioavailable fractions and sequestration mechanisms were manually curated from primary literature. This fraction is an operational correction for cross-hormone scaling and does not capture tissue-specific uptake or local metabolism.

### Circulating half-life curation

Hormone half-lives were curated from primary literature with preference for values measured in healthy adults and for the active (and when possible unbound) circulating species. Candidate values were identified via systematic PubMed searches using AI-assisted literature screening (GPT-4.5 and GPT 5.1, OpenAI) with a structured extraction protocol specifying inclusion criteria (human adults, healthy/fasting baseline, IV or infusion pharmacokinetic studies, terminal elimination phase), exclusion criteria (pediatric, pregnancy, disease states, synthetic analogs), and required output fields including the exact location of the extracted value within each source paper. All retrieved values were manually verified against original publications at the reported location before inclusion in S2 Data. Where human data were unavailable, rodent studies were included with clear species annotation. When multiple kinetic phases were reported, we used the terminal (β-phase) half-life as a summary of systemic clearance, and retained notes about the experimental context (endogenous decay, bolus injection, or infusion) and assay format. Reported half-lives were converted to seconds; when studies reported a range or confidence interval, we retained lower and upper bounds and used a central estimate (geometric mean when bounds were multiplicative) for analyses requiring a single value. Primary clearance mechanisms were annotated when reported in the source.

### Quality control, independence of analysis, and roles

Affinity, bioavailability, and half-life subtables were assembled by physician curators (T.K. and S.B.) under supervision of S.F. Each entry was checked for unit consistency, molecular form (total versus free), and alignment between the assay readout and the curated parameter. S.F. reviewed the completed tables, resolved discrepancies across sources, and approved finalized values prior to downstream modeling. The finalized master table was then provided to R.S., who performed the statistical modeling and figure generation independently of the curation process, using the fixed table as input and without iterative tuning of curated parameters.

AI-assisted tools (GPT-4, ChatGPT 5.0, OpenAI; Claude, Anthropic) were used during this work in two capacities: (i) preliminary literature search and data extraction during curation of affinity, bioavailability, and half-life datasets, and review of hormone list designations—all AI-retrieved values and classifications were manually verified against original publications before inclusion; and (ii) review of manuscript text for clarity and flow, with all final phrasing decisions made by the authors.

### Sensitivity analysis

DHEAS receptor assignment represents a source of uncertainty in the regression model, as DHEAS-specific receptors were only recently characterized. We assigned DHEAS to the sigma-1 receptor based on cryo-EM evidence for sulfate-selective binding [27, 38], but an alternative DHEAS-specific Gnα11-coupled GPCR with documented function at physiological concentrations was also identified [39]. We restricted our search to receptors for which DHEAS-specific binding or signaling has been demonstrated without requiring desulfation, which identified only these two candidates. To test whether the concentration–affinity correlation (Fig 4) depends on this assignment choice, we substituted a provisional Kd of 500 nM (reflecting the functional affinity range of the Gnα11-GPCR) for the sigma-1 Kd (18 µM) and re-ran the log–log regression on the 56-hormone dataset. *R*^2^ decreased from 0.31 to 0.26 (*p* = 5.1 × 10^−5^). The concentration–affinity correlation persisted across both DHEAS-specific receptor assignments.

### Visualization and data analysis

We generated distribution maps of both circulating hormone mass and the number of circulating hormone molecules using the Bionic Visualizations (Proteomaps) tool (http://bionic-vis.biologie.uni-greifswald.de/index.php). For the affinity analyses, we fit linear regression models in log10 space relating binding affinity (log10 Kd) to circulating concentration (total or receptor-available). We evaluated extensions that included half-life as a covariate and models allowing slopes and intercepts to vary by hormone class; nested model comparisons used *F*-tests and information criteria, and uncertainty was summarized with 95% confidence intervals from predictive distributions. Full model specifications, tests, and software libraries are provided in the S1 Appendix. For bioavailability analyses, we used a *χ*^2^ test of independence to assess the association between sequestration mechanism and hormone class, with effect size quantified by Cramér’s V. Differences in receptor-available fraction across classes were tested by one-way ANOVA with Tukey HSD post-hoc comparisons. The relationship between receptor-available fraction and circulating half-life among sequestered hormones was assessed by linear regression in log_10_ space. These tests were performed in Python using scipy.stats. Code used to generate Figs 3–5 and Figs E, H, and I in S1 Appendix is available in the public GitLab repository (tagged release v1.0.0) and archived on Zenodo (DOI: https://doi.org/10.5281/zenodo.20110628) (see Data sharing statement).

### Precision evaluation and variability assessment

To evaluate the precision of our estimates and assess variability in the healthy population, we focused on DHEAS and adiponectin, the two most abundant hormones by copy number and mass. We systematically reviewed literature sources for individuals aged 20–30 years, extracting hormone level distributions. Discrete distributions were constructed from reported individual values, while normal or lognormal distributions were derived from summary statistics provided in the source analyses. Using bootstrapping techniques (1,000 iterations), we determined the median and its 95% confidence interval for each distribution, stratified by hormone and sex. Overall variation was assessed using the 5th and 95th percentiles of the aggregated distributions.

### Biological variation (CVi and CVg)

To contextualize biological variability in circulating hormone concentrations, we queried the EFLM Biological Variation Database [19] (accessed July 30, 2025) for hormones with available standardized within-subject (CVi) and between-subject (CVg) coefficients of variation. For each hormone, we recorded the reported CVi and CVg values and mapped hormones to the biochemical classes used throughout the manuscript. We summarized variability across hormones using medians and report hormone-specific CVi/CVg values in Table D in S1 Appendix. Hormones with pronounced circadian or pulsatile secretion were not modeled with time-resolved profiles; instead, the main analyses represent concentrations using reference-range–anchored summary values (geometric means), with temporal dynamics treated as contributing to dispersion within reported intervals.

### Comprehensive hormone list compilation

To evaluate the comprehensiveness of our 63 clinically-assayed hormones, we compiled an extensive list of human hormones. Sources included textbooks [16–18] and published repositories: the HORDB peptide hormone database [6] (5,729 entries), and the UniProt database [40] (queried on Aug-24–2023 for “hormone” AND “model organism 96 (Homo sapiens)”). We manually curated the resulting 2,267 entries, focusing on endogenous human endocrine hormones. Exclusion criteria are detailed in Figs F and G in S1 Appendix and corresponding tables. For the curated list of primary endocrine signaling molecules, we searched PubMed for serum/blood levels, converted results to pg/ml, and calculated total circulating mass for hormones not in our primary analysis (Table B in S1 Appendix). These results contribute to our error estimate.

## Supporting information

S1 Data Hormone distribution and circulating abundance estimates (Excel workbook). Workbook used to harmonize adult reference intervals and published concentration data and to derive circulating pool metrics (molarity, total mass, and total molecule counts). Tabs: Hormone_abundance; Hormones_sorted; AU_conversion; LOD; Uniprot_query; HorDB_query; Hormones_comprehensive.
(XLSX)

S2 Data Curated receptor affinity, bioavailability, and half-life annotations (Excel workbook). Workbook used for affinity and bioavailability analyses, including the operational hormone–primary receptor mapping, curated Kd values, receptor-available fractions with sequestration mechanism labels, and circulating half-lives. Tabs: Readme; Master_table; Kd; Hormone_affinity_detailed; Bioavailability; Half_life; Copy_of_Hormone_abundance.
(XLSX)

S1 Appendix Statistical Analysis of Receptor Binding Affinity and Circulating Hormone Concentration. Detailed model specifications, statistical testing procedures, and software implementation details for the affinity analyses presented in the main text, including model comparison metrics (Table A in S1 Appendix). **Fig A.** The distribution of hormone mass in young adult males. **Fig B.** Circulating hormone mass distribution by secreting tissue/gland in males and females. **Fig C.** Circulating hormone molarity distribution by secreting tissue/gland. **Fig D.** Histograms of molecular weight (weighted by molecule count and by total mass). **Fig E.** Distribution of circulating hormones by molecular weight and abundance in males. **Fig F.** Distribution of candidate molecules in UniProt database. **Fig G.** Distribution of candidate molecules in HORDB databases. **Fig H.** Receptor affinity versus total circulating hormone molarity. Fig I. Sequestration mechanisms by hormone type. **Table A.** Model comparison metrics for affinity regression models (included in S1 Appendix). **Table B.** Suggested compilation of human hormones for clinical endocrinology. **Table C.** Collated adiponectin and DHEAS data from healthy young adult cohorts. **Table D.** Collated reference change values (RCVs) for 25 hormones [19,34,41–47].
(DOCX)

## Abbreviations

DHEAS: dehydroepiandrosterone sulfate
LOD: limit of detection
NIBSC: National Institute for Biological Standards and Control
