# Supplementary material for: The total mass, copy number, and distribution of hormones in the human bloodstream
Source: PLoS Biol. 2026 Jun 22;24(6):e3003864. doi: 10.1371/journal.pbio.3003864 (PMC13349298; doi:10.1371/journal.pbio.3003864)
Supplement: S1 Appendix — Detailed model specifications, statistical testing procedures, and software implementation details for the affinity analyses presented in the main text, including model comparison metrics (Table A in S1 Appendix). Fig A. The distribution of hormone mass in young adult males. Fig B. Circulating hormone mass distribution by secreting tissue/gland in males and females. Fig C. Circulating hormone molarity distribution by secreting tissue/gland. Fig D. Histograms of molecular weight (weighted by molecule count and by total mass). Fig E. Distribution of circulating hormones by molecular weight and abundance in males. Fig F. Distribution of candidate molecules in UniProt database. Fig G. Distribution of candidate molecules in HORDB databases. Fig H. Receptor affinity versus total circulating hormone molarity. Fig I. Sequestration mechanisms by hormone type. Table A. Model comparison metrics for affinity regression models (included in S1 Appendix). Table B. Suggested compilation of human hormones for clinical endocrinology. Table C. Collated adiponectin and DHEAS data from healthy young adult cohorts. Table D. Collated reference change values (RCVs) for 25 hormones [19,34,41–47]. (DOCX) [file pbio.3003864.s003.docx]

S1 Appendix. Statistical Analysis of Receptor Binding Affinity and Circulating Hormone Concentration

### Overview

This supplementary section provides detailed model specifications, statistical testing procedures, and software implementation details for the affinity analyses presented in the main text. We fit linear regression models in log₁₀ space relating binding affinity (log₁₀ Kd) to circulating concentration (total or receptor-available), evaluated extensions including half-life as a covariate, and tested models allowing slopes and intercepts to vary by hormone class.

## Data and Transformation

### Dataset

The analysis comprised 56 hormones with validated receptor binding affinity values. For each hormone, we used the binding affinity (Kd) for its primary physiological receptor. Hormones were classified into four types: Amine (n=7), Glycoprotein (n=7), Peptide (n=26), and Steroid (n=16).

## Concentration Measures

Two measures of circulating hormone concentration were considered:

1. Total molarity ($C_{total}$): The total circulating molar concentration
2. Free molarity ($C_{free}$): The bioavailable (unbound) fraction, calculated as:

$$C_{free}=C_{total}\times f_{free}$$

- where $f_{free}$ is the free fraction (proportion not bound to carrier proteins or otherwise sequestered)

### Log-Transformation

All regression analyses were performed in log₁₀ space. This transformation was used because both Kd values and concentrations span multiple orders of magnitude (10⁻¹³ to 10⁻⁵ M). Log-transformation linearizes power-law relationships of the form $K_{d}=A\cdot C^{\beta}$

## Model Specifications

We fitted a hierarchy of nested linear regression models with increasing complexity.

### Univariate Models

1. Total Molarity: ${log}_{10}(K_{d})=\beta_{0}+\beta_{1}{log}_{10}(C_{total})+\varepsilon$
2. Free Molarity: ${log}_{10}(K_{d})=\beta_{0}+\beta_{1}{log}_{10}(C_{free})+\varepsilon$

where $\varepsilon\sim N(0,\sigma^{2})$ represents independent, identically distributed errors.

### Bivariate Models with Half-Life

1. Free Molarity + Half-life (Additive):

$${log}_{10}(K_{d})=\beta_{0}+\beta_{1}{log}_{10}(C_{free})+\beta_{2}{log}_{10}(t_{1/2})+\varepsilon$$

1. Free Molarity + Half-life + Interaction:

$${log}_{10}(K_{d})=\beta_{0}+\beta_{1}{log}_{10}(C_{free})+\beta_{2}{log}_{10}(t_{1/2})+\beta_{3}\left[ {log}_{10}(C_{free})\cdot{log}_{10}(t_{1/2}) \right]+\varepsilon$$

### Hormone Type Models

1. Free Molarity by Hormone Type (Varying Slopes and Intercepts):

$${log}_{10}(K_{d})=\beta_{0}+\sum_{j=2}^{4} \delta_{j}T_{j}+\beta_{1}{log}_{10}(C_{free})+\sum_{j=2}^{4} \gamma_{j}\left[ T_{j}\cdot{log}_{10}(C_{free}) \right]+\varepsilon$$

where:

- $T_{j}$ are indicator (dummy) variables for hormone types j = 2 (Glycoprotein), 3 (Peptide), 4 (Steroid)
- Amine serves as the reference category (j = 1)
- $\delta_{j}$ represents the difference in intercept for type j relative to Amine
- $\gamma_{j}$ represents the difference in slope for type j relative to Amine
- The slope for Amine is $\beta_{1}$; for other types it is $\beta_{1}+\gamma_{j}$

This parameterization allows each hormone type to have a distinct power-law relationship:

$$K_{d}=A_{j}\cdot C_{free}^{\beta_{j}}$$

where $A_{j}={10}^{\beta_{0}+\delta_{j}}$ and $\beta_{j}=\beta_{1}+\gamma_{j}$ for each type j.

### Extended Models with Half-Life

1. Model 5 + Half-life (Main Effect Only):

$${log}_{10}(K_{d})=\beta_{0}+\sum_{j=2}^{4} \delta_{j}T_{j}+\beta_{1}{log}_{10}(C_{free})+\sum_{j=2}^{4} \gamma_{j}\left[ T_{j}\cdot{log}_{10}(C_{free}) \right]+\beta_{2}{log}_{10}(t_{1/2})+\varepsilon$$

1. Model 6 + Concentration × Half-life Interaction:
   ${log}_{10}(K_{d})=\beta_{0}+\sum_{j=2}^{4} \delta_{j}T_{j}+\beta_{1}{log}_{10}(C_{free})+\sum_{j=2}^{4} \gamma_{j}\left[ T_{j}\cdot{log}_{10}(C_{free}) \right]+\beta_{2}{log}_{10}(t_{1/2})+$

$$\beta_{3}\left[ {log}_{10}(C_{free})\cdot{log}_{10}(t_{1/2}) \right]+\varepsilon$$

1. Model 5 + Half-life × Hormone Type Interactions Adds half-life with type-specific slopes:

$${log}_{10}(K_{d})=\beta_{0}+\sum_{j=2}^{4} \delta_{j}T_{j}+\beta_{1}{log}_{10}(C_{free})+\sum_{j=2}^{4} \gamma_{j}\left[ T_{j}\cdot{log}_{10}(C_{free}) \right]+\beta_{2}{log}_{10}(t_{1/2})$$

$$+\sum_{j=2}^{4} \phi_{j}\left[ T_{j}\cdot{log}_{10}(t_{1/2}) \right]+\varepsilon$$

## Statistical Testing

### Overall Model Significance (F-test)

The null hypothesis that all regression coefficients equal zero was tested using the F-statistic:

$$F=\frac{{R^{2}}/k}{(1-R^{2})/(n-k-1)}$$

where:

- $R^{2}$ is the coefficient of determination
- $k$ is the number of predictors (excluding intercept)
- $n$ is the sample size

Under the null hypothesis, F follows an F-distribution with $(k,n-k-1)$ degrees of freedom. A p-value < 0.05 was considered statistically significant.

### Individual Coefficient Significance (t-tests)

The significance of individual regression coefficients was assessed using t-statistics:

$$t=\frac{\hat{\beta}_{i}}{SE(\hat{\beta}_{i})}$$

where the standard error is derived from the variance-covariance matrix of the coefficients:

$$Var(\hat{\beta})=\hat{\sigma}^{2}(X^{T}X)^{-1}$$

with $\hat{\sigma}^{2}=MSE=\frac{1}{n-k-1}\sum_{i=1}^{n} (y_{i}-\hat{y}_{i})^{2}$.

The t-statistic follows a t-distribution with $(n-k-1)$ degrees of freedom under the null hypothesis $H_{0}:\beta_{i}=0$.

### Nested Model Comparisons

For comparing nested models, we used multiple criteria:

Adjusted R²: Penalizes additional parameters to prevent overfitting:

$$R_{adj}^{2}=1-(1-R^{2})\frac{n-1}{n-k-1}$$

Akaike Information Criterion (AIC):

$$AIC=2k-2ln(\hat{L})$$

For linear regression with normally distributed errors:

$$AIC\approx nln\left( \frac{RSS}{n} \right)+2(k+1)$$

where RSS is the residual sum of squares and we include the variance parameter in the count.

Lower AIC values indicate better model fit relative to complexity. BIC imposes a stronger penalty for additional parameters when $n>8$.

## Uncertainty Quantification

### Confidence Intervals for the Regression Line

95% confidence intervals for the mean predicted value at a given concentration were computed using the hat matrix formulation:

$$\hat{y}(x_{0})\pm t_{0.975,n-k-1}\cdot SE_{mean}(x_{0})$$

where:

$$SE_{mean}(x_{0})=\sqrt{MSE\cdot h_{00}}$$

and $h_{00}=x_{0}^{T}(X^{T}X)^{-1}x_{0}$ is the leverage of the prediction point.

For multivariate models, $x_{0}$ includes all features (log-concentration, hormone type dummies, and interaction terms) appropriately coded for the target hormone type.

### Prediction Intervals

Prediction intervals for a new individual observation would additionally include the residual variance:

$$SE_{pred}(x_{0})=\sqrt{MSE\cdot(1+h_{00})}$$

### Interpretation

Confidence intervals were computed in log₁₀ space and back-transformed to the original scale. Because the transformation is monotonic, the resulting intervals maintain their coverage properties.

## Software Implementation

All analyses were performed using Python 3.13.7 with the following libraries:

| **Library** | **Version** | **Purpose** |
| --- | --- | --- |
| scikit-learn | 1.7.2 | Linear regression fitting, R² computation via .score() method |
| scipy | 1.16.2 | Statistical distributions (F, t), p-value calculations |
| numpy | 2.3.3 | Linear algebra operations, matrix computations for standard errors |
| pandas | 2.3.3 | Data manipulation, feature matrix construction, dummy variable encoding |
| matplotlib | 3.10.7 | Figure generation |
| seaborn | 0.13.2 | Statistical visualization, regression plots |

### Implementation Details

- Regression fitting: sklearn.linear_model.LinearRegression with default parameters (ordinary least squares)
- Dummy variable encoding: pandas.get_dummies() with drop_first=True to avoid multicollinearity
- Standard error calculation: Custom implementation using $(X^{T}X)^{-1}$ via numpy.linalg.inv()
- Critical values: scipy.stats.t.ppf() for t-distribution, scipy.stats.f.cdf() for F-distribution

## Model Performance Summary

| Model | Description | k | R² | Adj. R² | RMSE | AIC |
| --- | --- | --- | --- | --- | --- | --- |
| 1 | Total Molarity | 1 | 0.290 | 0.276 | 1.03 | 166.5 |
| 2 | Free Molarity | 1 | 0.306 | 0.293 | 1.02 | 165.2 |
| 3 | Free Molarity + Halflife | 2 | 0.290 | 0.264 | 1.04 | 168.5 |
| 4 | Free Molarity + Halflife + Interaction | 3 | 0.291 | 0.250 | 1.05 | 170.3 |
| 5 | Free Molarity by Hormone Type | 7 | 0.462 | 0.384 | 0.95 | 162.9 |
| 6 | Model 5 + Halflife (main) | 8 | 0.465 | 0.368 | 0.96 | 164.9 |
| 7 | Model 5 + Halflife × Conc. | 9 | 0.467 | 0.352 | 0.97 | 166.8 |
| 8 | Model 5 + Halflife × Type | 11 | 0.474 | 0.341 | 0.98 | 169.2 |

Table A. Model comparison metrics. k = number of predictors (excluding intercept); RMSE = root mean squared error in log₁₀ units; n = 56 for all models. Model 5 achieved the lowest AIC and highest adjusted R², indicating the best balance between fit and parsimony. Adding half-life (Models 6-8) did not improve model fit, as indicated by higher AIC values and lower adjusted R².

## Model Selection Rationale

Model 5 (free molarity with hormone type-specific slopes and intercepts) was selected as the preferred model based on:

1. Lowest AIC (162.9): Best trade-off between goodness-of-fit and complexity
2. Highest Adjusted R² (0.384): Explains the most variance after penalizing for parameters
3. Significant overall F-test (p < 0.001): The model explains significantly more variance than a null model
4. Biological interpretability: Allows different hormone classes to exhibit distinct concentration-affinity relationships, consistent with their different receptor architectures and signaling mechanisms

The addition of half-life as a covariate (Models 3-4, 6-8) did not improve model fit in this linear framework, as evidenced by:

- Increased AIC in all half-life models
- Decreased adjusted R² relative to the simpler Model 5
- Non-significant coefficients for half-life terms

This does not exclude more complex, non-linear relationships between half-life and receptor affinity, but suggests that within this linear modeling framework, hormone concentration and type capture the primary sources of variation in receptor binding affinity.

# Supplementary figures and tables

###
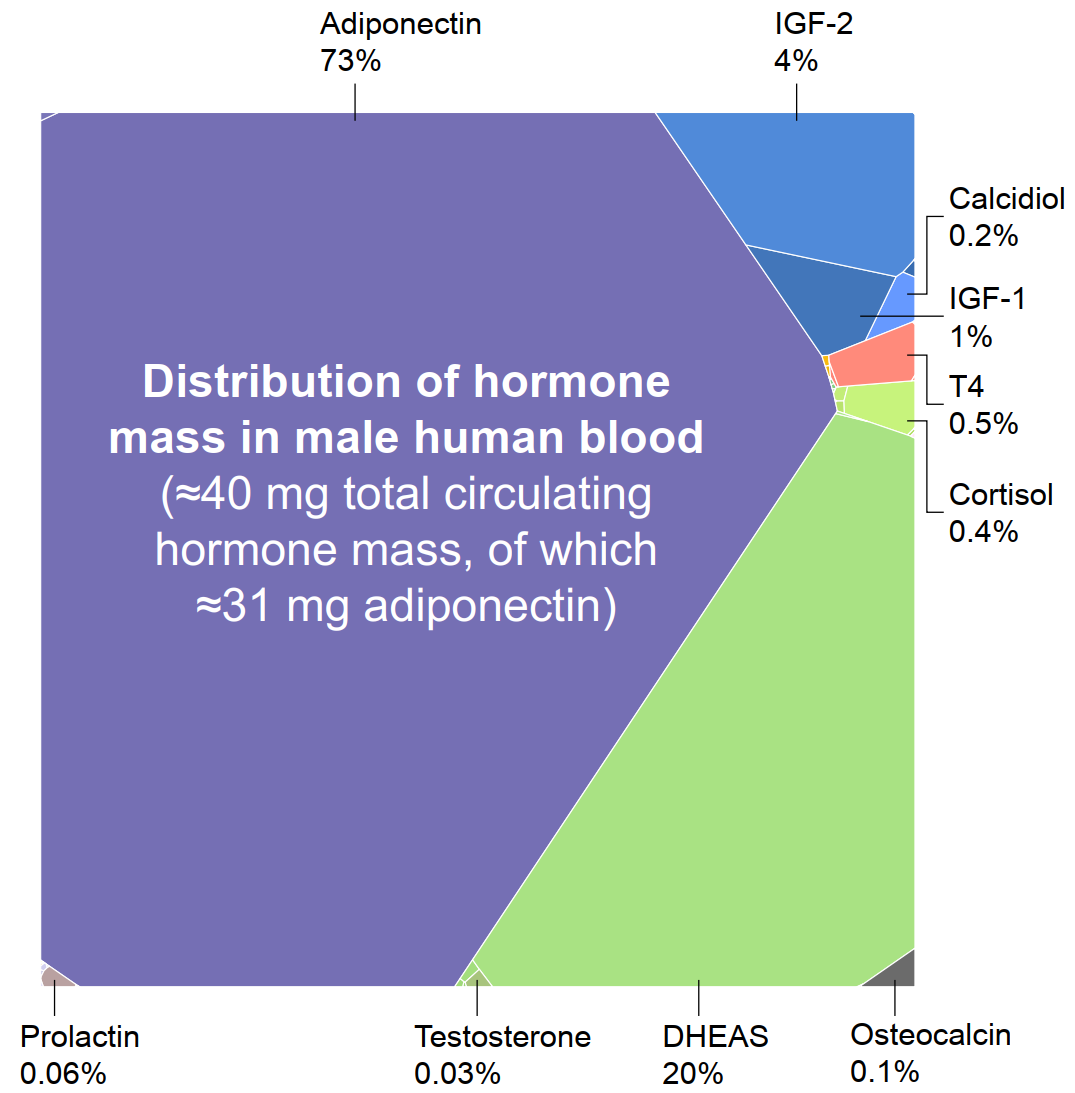


### Fig A. The distribution of hormone mass in young adult males.

Voronoi diagrams illustrate the distribution of the circulating hormones by mass in men (see figure 1 for women). The area of each polygon is proportional to the relative mass of each hormone. Colors represent different endocrine systems or hormone types. The top 10 contributors are labeled with their names and percentage contributions. In men the contribution of adiponectin is smaller while DHEAS greater than in women. Total circulating hormone mass is approximately 40 mg in both women and men. Underlying data: S1 Data, sheet Hormone_abundance. Generated using the Proteomaps tool (http://bionic-vis.biologie.uni-greifswald.de/).

###

###


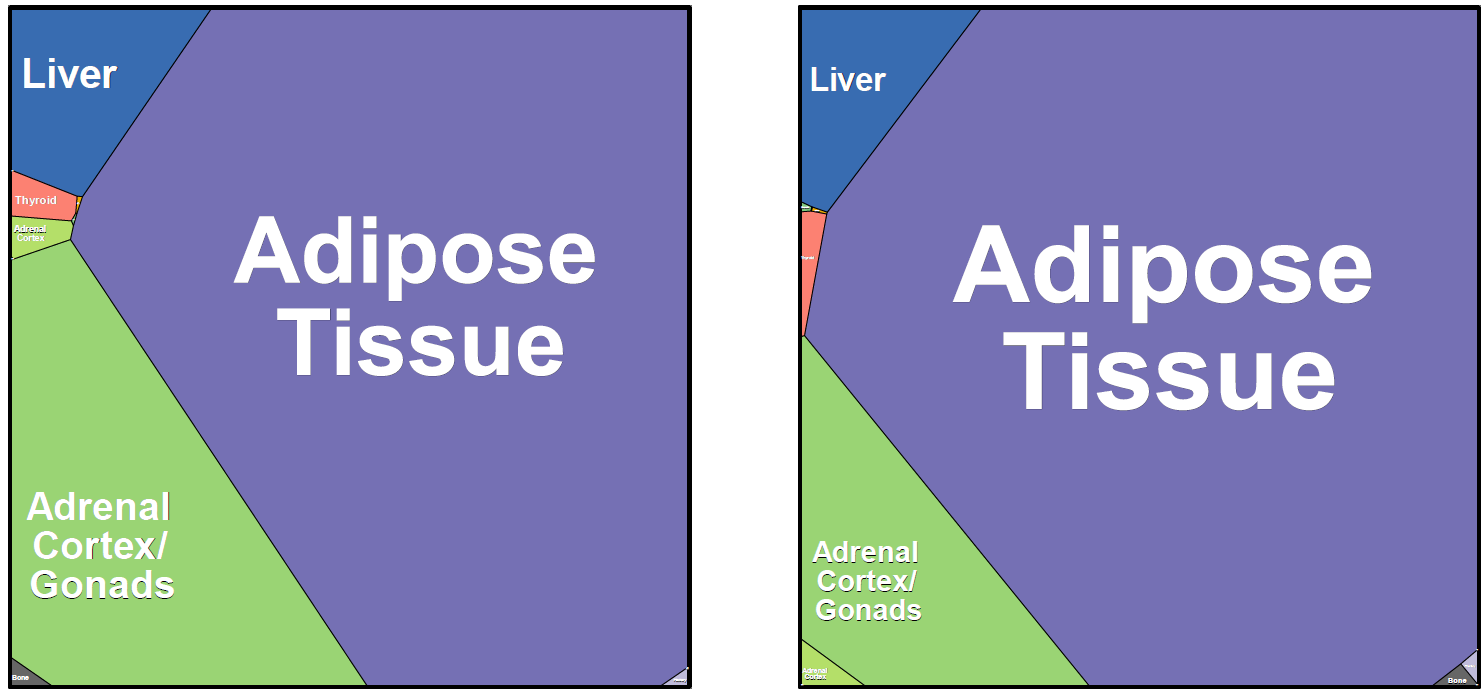


###

### Fig B. Circulating hormone mass distribution by secreting tissue/gland in males and females. Voronoi diagrams illustrate the distribution of circulating hormones by mass in males (left) and females (right). The area of each polygon is proportional to the relative mass of hormones secreted by each tissue/gland. Colors represent different secreting tissues/glands as in Fig 1. Adipose tissue dominates hormone mass in both sexes, primarily due to adiponectin. Together with the liver we note the dominant mass contribution of hormones derived from non-gland organs. Underlying data: S1 Data, sheet Hormone_abundance. Generated using the Proteomaps tool (http://bionic-vis.biologie.uni-greifswald.de/).

###
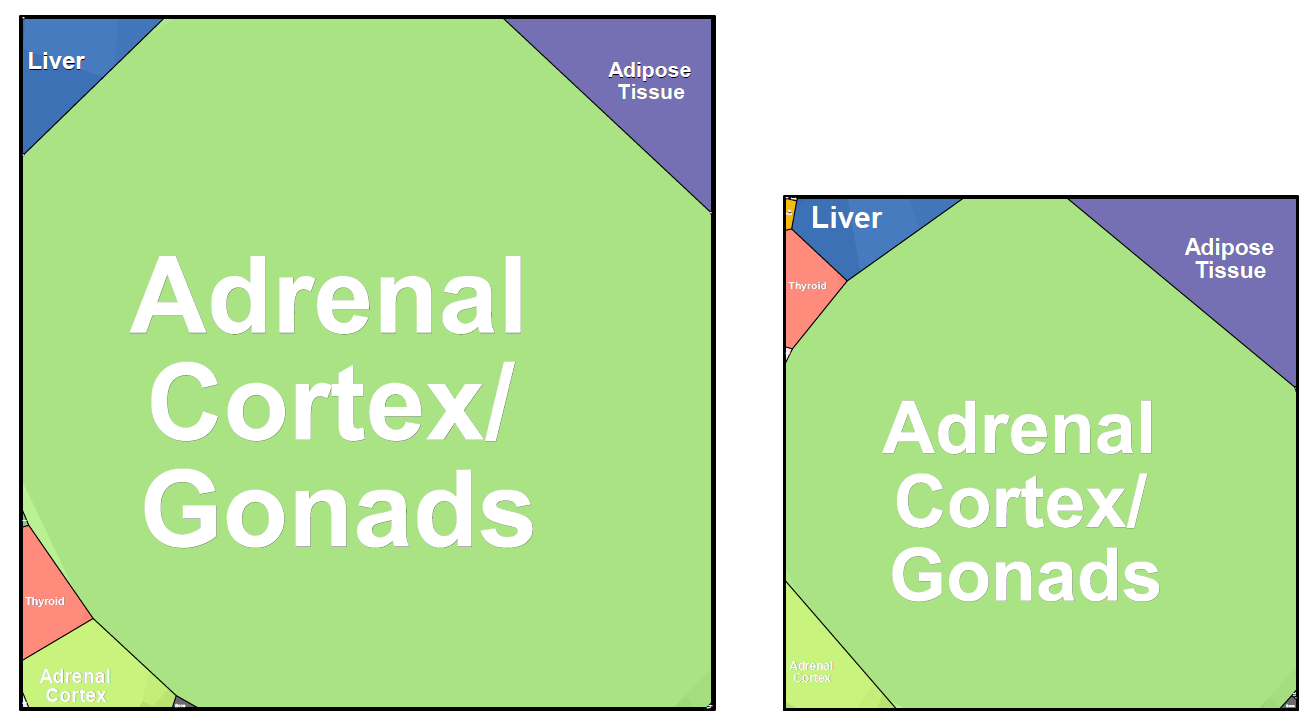


### Fig C. Circulating hormone molarity distribution by secreting tissue/gland. The distribution is represented by Voronoi diagrams, such that the area of each polygon is proportional to the fraction of total circulating hormone molecules secreted by each tissue/gland in males (left) and females (right). Colors represent different secreting tissues/glands as in Fig 1. The adrenal cortex/gonads dominate the landscape in both sexes, primarily due to DHEAS, constituting approximately 90% of circulating hormone molecules. The male diagram (left) is larger, reflecting the higher total number of circulating hormone molecules in males (≈25 μmol) compared to females (≈14 μmol). This visualization highlights the contrast between hormone mass (Fig A) and molecule count distributions, emphasizing the dominant role of adrenal-derived steroids in the molecular composition of the circulating hormone repertoire. Underlying data: S1 Data, sheet Hormone_abundance. Generated using the Proteomaps tool (http://bionic-vis.biologie.uni-greifswald.de/).

###


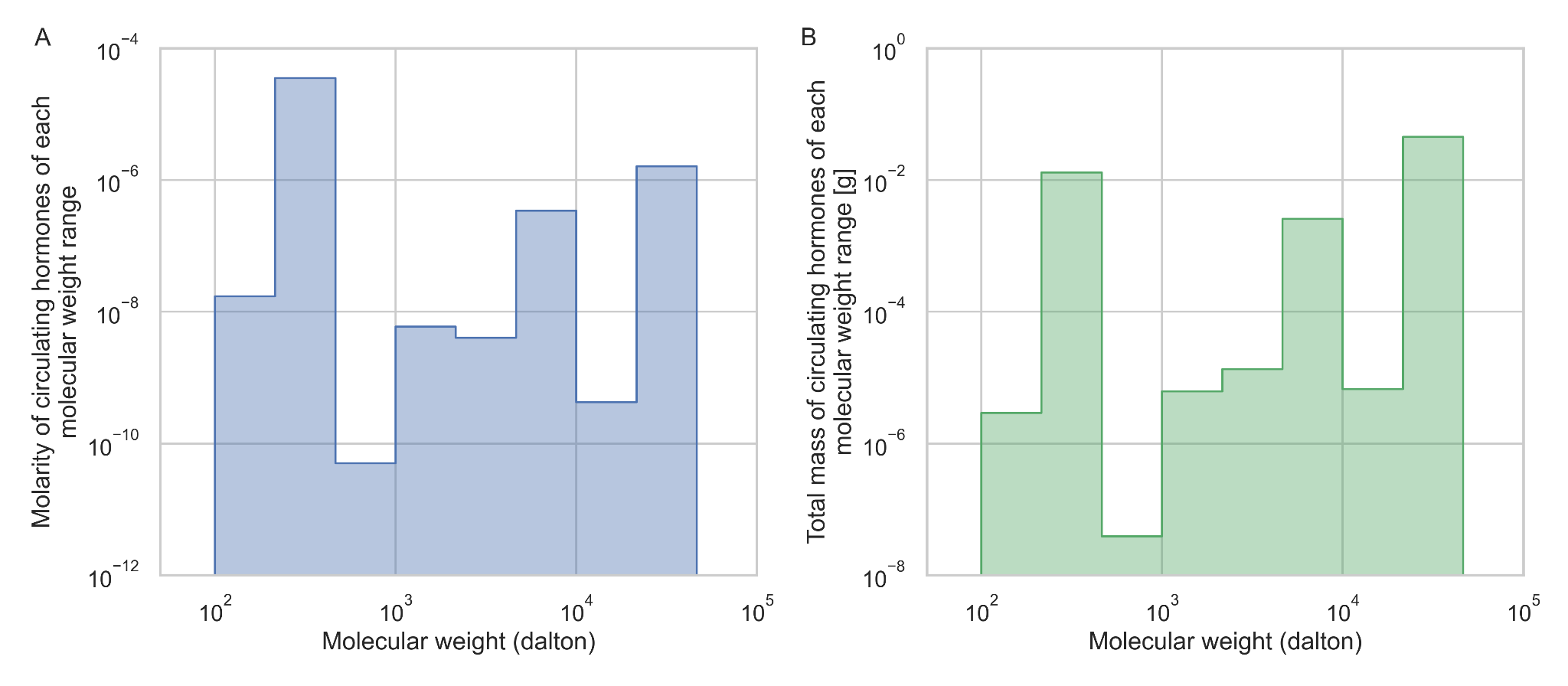


###

###

### Fig D. Histograms of the molecular weight.

*A.* Molecular weight, weighted by the number of molecules of the circulating hormones**.** *Circulating hormones were binned according to their molecular weight. For each bin the total molarity of hormones within the bin was then calculated and presented.*

*B.* Molecular weight, weighted by the total mass of the circulating hormones**.** *Circulating hormones were binned according to their molecular weight. For each bin the total mass of hormones within the bin was then calculated and presented.* Underlying data: S1 Data, sheet Hormone_abundance.

###
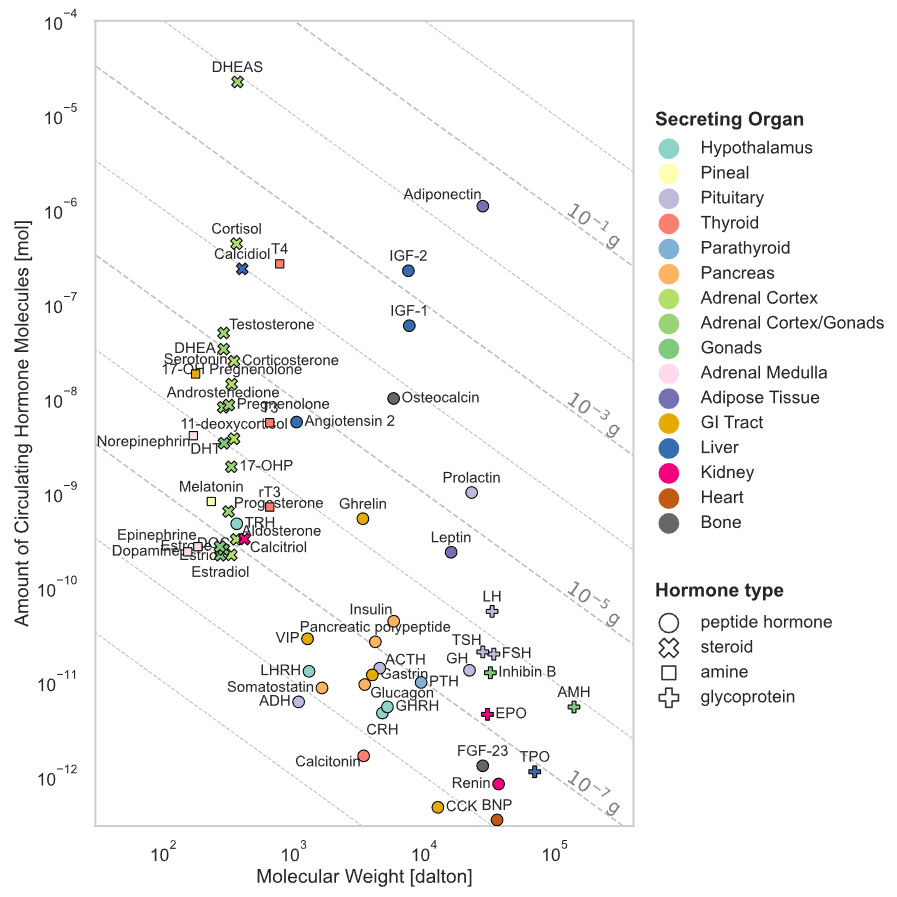


### Fig E. Distribution of circulating hormones by molecular weight and abundance in males. Hormone molecules are plotted by molecular weight (x-axis) and circulating abundance (y-axis). Colors indicate the secreting organ, and shapes denote hormone types (e.g., peptide, steroid, amine, glycoprotein) as shown in the legend. Diagonal iso-mass lines represent the total circulating mass in grams (given by the product of the two axes). This figure for males complements Fig 3 in the main text, which shows data for females. Underlying data: S1 Data, sheet Hormone_abundance. Code: Zenodo DOI 10.5281/zenodo.20110628.


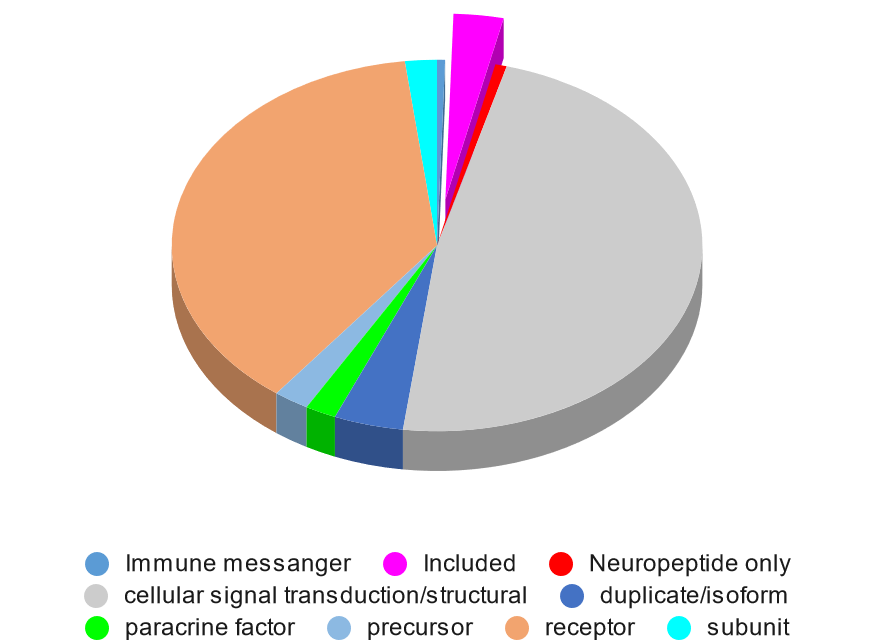


###

|  | **count** |
| --- | --- |
| **hormones included** | 68 |
| immune messenger | 11 |
| neuropeptide only | 17 |
| cellular signal transduction/structural | 1085 |
| duplicate/isoform | 94 |
| paracrine factor | 42 |
| precursor | 52 |
| receptor | 855 |
| subunit | 44 |
| **total** | **2267** |

###

###

### Fig F. Distribution of candidate molecules in Uniprot database

The pie chart represents manual curation of a search for hormones in homo-sapiens in the Uniprot database. The search returned 2267 entries, of which 68 (3%) were found to be hormones and included in the hormone list (labeled “Included”). Causes of exclusion are listed along with the number of entries per each (S1 Data, sheet: Uniprot query).


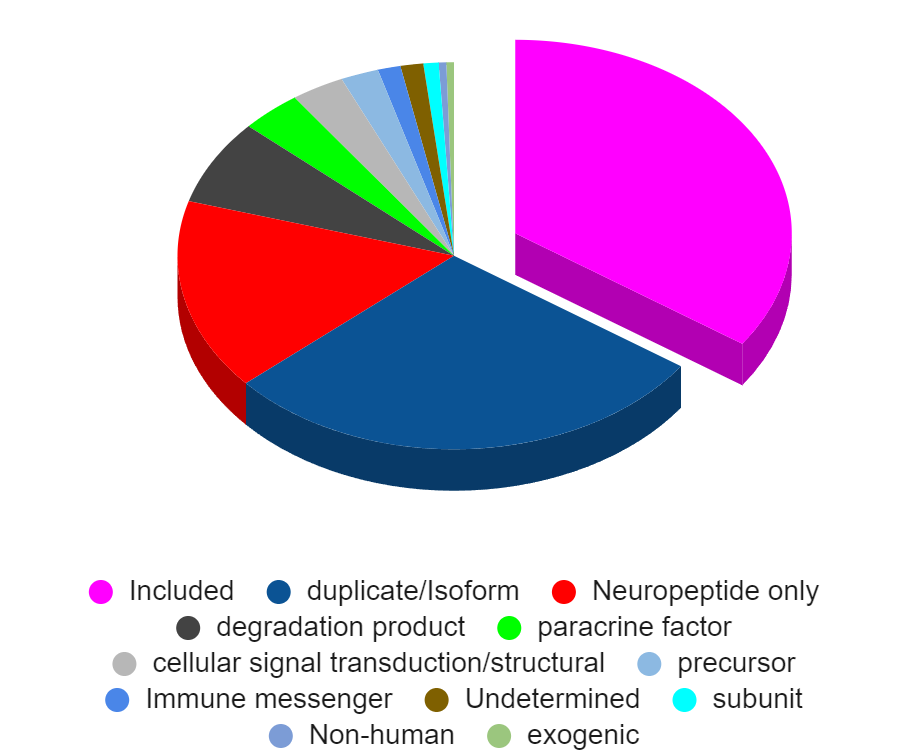


|  | **count** | **Fraction** |
| --- | --- | --- |
| **hormones included** | **72** | **32.0%** |
| duplicate/Isoform | 63 | **28.0%** |
| neuropeptide only | 39 | **17.3%** |
| degradation product | 16 | **7.1%** |
| paracrine factor | 8 | **3.6%** |
| precursor | 8 | **3.6%** |
| cellular signal transduction/structural | 9 | **4.0%** |
| Immune messenger | 3 | **1.3%** |
| Undetermined | 3 | **1.3%** |
| subunit | 2 | **0.9%** |
| Non-human | 1 | **0.4%** |
| exogenic | 1 | **0.4%** |
| **total** | **225** |  |

### Fig G. Distribution of candidate molecules in HORDB databases

The pie chart represents a classification of peptides from the HORDB, a detailed peptide hormone database. Out of 5,729 HORDB entries reviewed by organisms, 225 (3.9%) human-expressed peptides were further curated. Among these, 75 peptides (33.3%) were confirmed as hormones and subsequently incorporated into the hormone list, denoted as “Included”. The chart also details reasons for exclusion with corresponding entry counts (S1 Data, sheet: HorDB query).


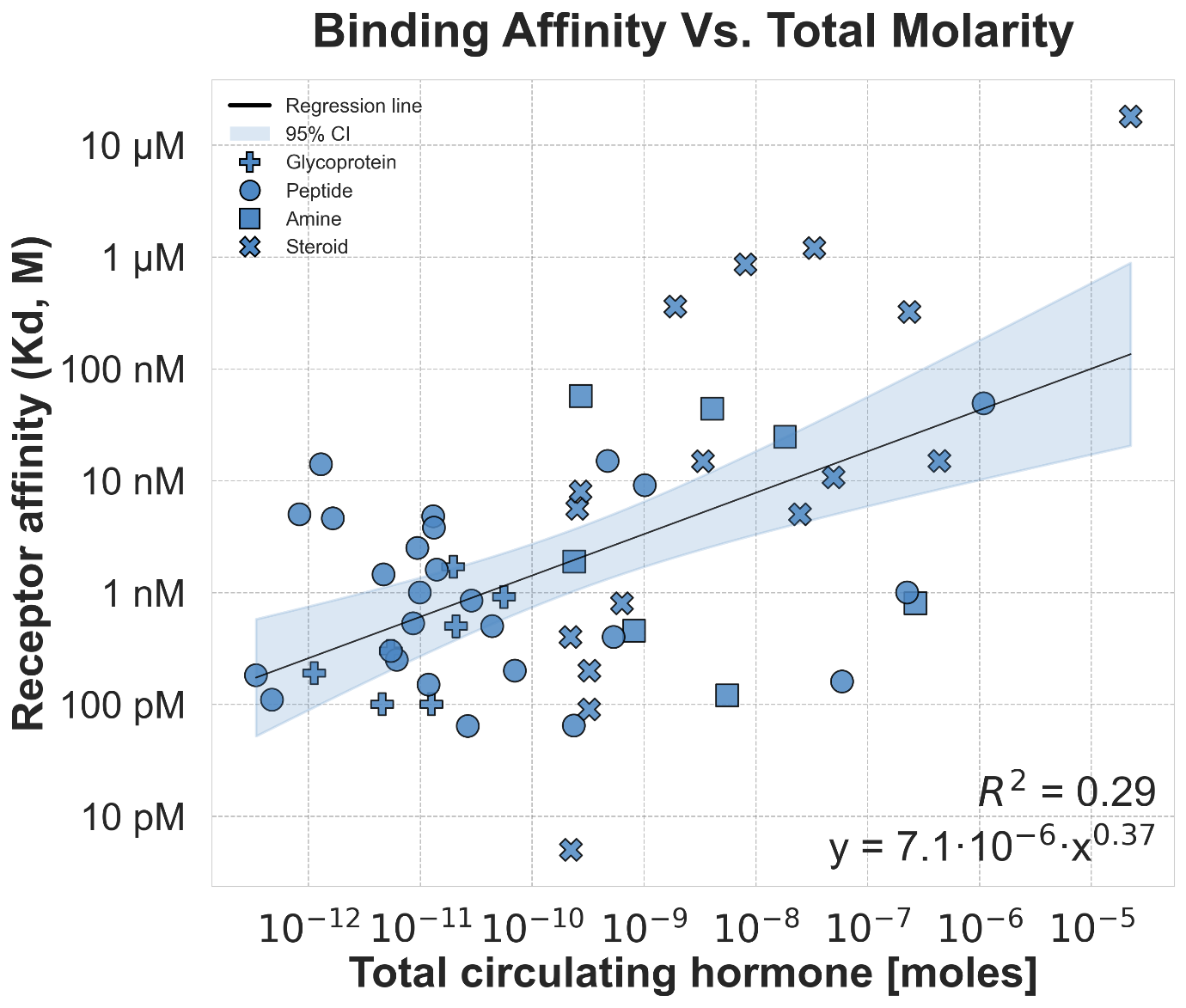


**Fig H. Receptor affinity versus total circulating hormone molarity**. Scatterplot of receptor binding affinity (equilibrium dissociation constant, Kd; y-axis) against total circulating hormone molarity (x-axis) across hormone–receptor pairs (n = 56). Axes are log-scaled; points are colored by hormone class (steroid, peptide, glycoprotein, amine). Solid line shows the least-squares fit in log–log space with shaded 95% confidence interval. Model summary is shown in the inset (R² = 0.29; adj. R² = 0.28; F = 22.1; p < 0.001), along with the fitted power-law relationship. Underlying data: S2 Data, sheets Master_table and Kd. Code: Zenodo DOI 10.5281/zenodo.20110628.


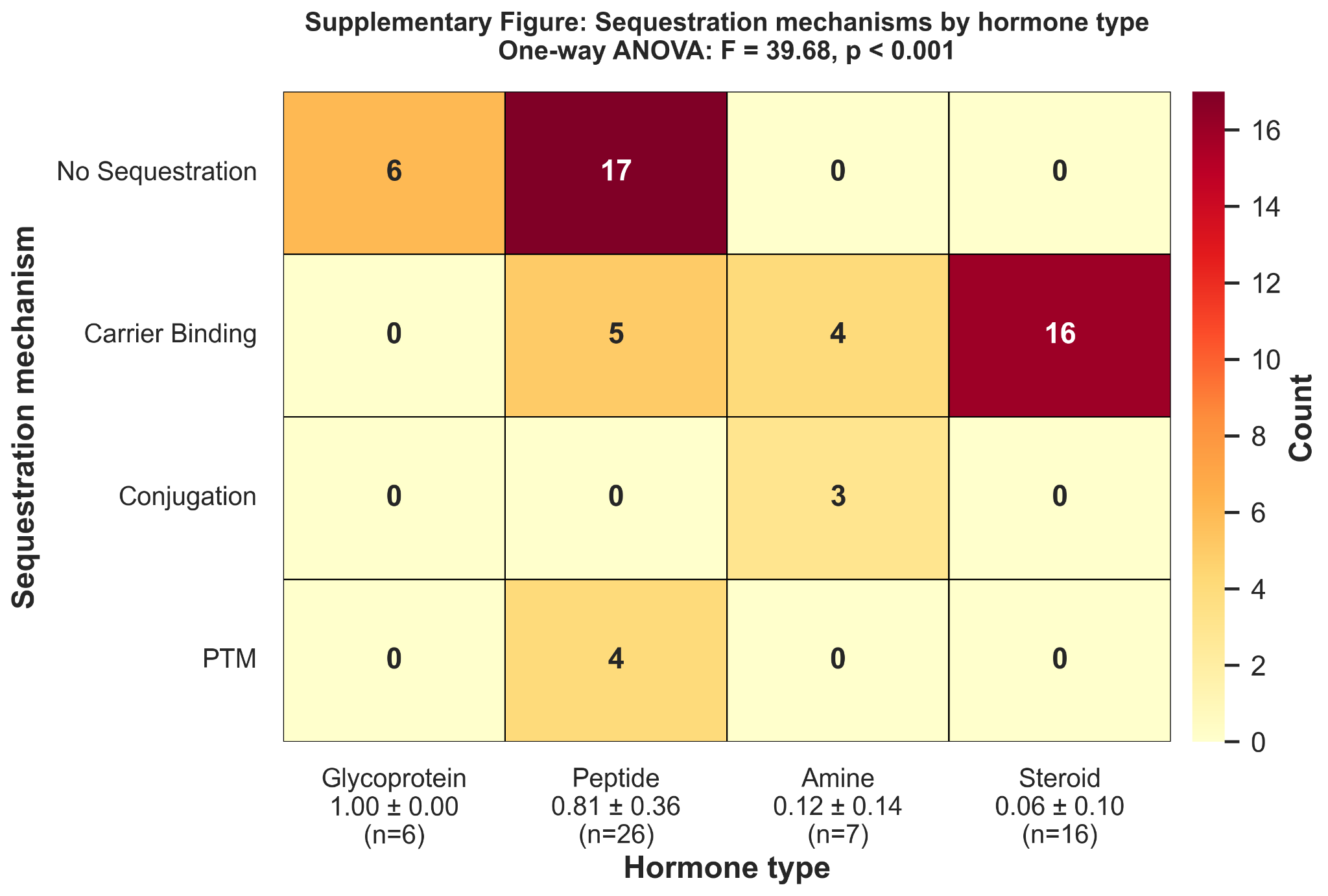


**Fig I. Sequestration mechanisms by hormone type**

Heatmap showing the number of hormones in each sequestration category (rows) across hormone types (columns). Cell values are counts; color intensity reflects count (scale at right). Sequestration mechanism is associated with hormone type (χ² = 60.31, p < 0.001, Cramér’s V = 0.61). Mean ± SD receptor-available fraction and sample size (n) for each hormone type are shown below the column labels. Sequestration categories: no sequestration, carrier binding, conjugation, and post-translational modification (PTM). Mean ± SD receptor-available fraction for each hormone type shown below column labels (with sample size n). Underlying data: S2 Data, sheets Master_table and Bioavailability. Code: Zenodo DOI 10.5281/zenodo.20110628.

| **Serial** | **Hormone name** |
| --- | --- |
| 1 | 11 beta-hydroxyandrostenedione (11OHA4) |
| 2 | 11-deoxycorticosterone (DOC) |
| 3 | 11-deoxycortisol |
| 4 | 11-ketoandrostenedione (11KA4) |
| 5 | 11-ketotestosterone (11KT) |
| 6 | 17-OH Pregnenolone |
| 7 | 17-OH Progesterone |
| 8 | 17-OH pregnenolone sulfate |
| 9 | 18-hydroxycorticosterone |
| 10 | 21-Deoxycortisol |
| 11 | Activin |
| 12 | Adiponectin |
| 13 | Adrenaline (epinephrine) |
| 14 | Adrenocorticotropic hormone (ACTH) |
| 15 | Adrenomedullin |
| 16 | Adropin |
| 17 | Agouti-related peptide (AgRP) |
| 18 | Alarin |
| 19 | Aldosterone |
| 20 | Alpha-neoendorphin |
| 21 | Amphiregulin |
| 22 | Androstenediol |
| 23 | Androstenediol 3-sulfate |
| 24 | Androstenedione |
| 25 | Androsterone |
| 26 | Androsterone sulfate |
| 27 | Angiotensin II |
| 28 | Angiotensin III |
| 29 | Angiotensin IV |
| 30 | Anti-diuretic hormone (ADH) |
| 31 | Anti-Mullerian hormone |
| 32 | Apelin |
| 33 | Arginine vasopressin |
| 34 | Asprosin |
| 35 | Atrial natriuretic peptide (ANP) |
| 36 | Augurin |
| 37 | beta-endorphin |
| 38 | Bradykinin |
| 39 | Brain natriuretic peptide (BNP) |
| 40 | Brain-derived neurotrophic factor (BDNF) |
| 41 | C-type natriuretic peptide (CNP) |
| 42 | Calcidiol |
| 43 | Calcitonin |
| 44 | Calcitonin gene-related peptide 1 (CGRP-1) |
| 45 | Calcitriol |
| 46 | Cardiotrophin-1 (CT-1) |
| 47 | Chemerin |
| 48 | Cholecystokinin (CCK) |
| 49 | Chorionic gonadotropin (hCG) |
| 50 | Chorionic somatomammotropin hormone 1 |
| 51 | Chorionic somatomammotropin hormone 2 |
| 52 | Chorionic somatomammotropin hormone like 1 |
| 53 | Corticosterone |
| 54 | Corticotropin-releasing hormone (CRH) |
| 55 | Cortisol |
| 56 | Cortisone |
| 57 | cortistatin |
| 58 | Dehydroepiandrosterone (DHEA) |
| 59 | Dehydroepiandrosterone-sulfate (DHEAS) |
| 60 | Dihydrotestosterone (DHT) |
| 61 | Dihydrotestosterone-sulfate (DHT-S) |
| 62 | Dopamine |
| 63 | Dopamine-sulfate |
| 64 | Dynorphin A |
| 65 | Dynorphin B |
| 66 | Elabela |
| 67 | Endothelin-1 |
| 68 | Endothelin-2 |
| 69 | Endothelin-3 |
| 70 | epiandrosterone |
| 71 | Erythropoietin |
| 72 | Estetrol (E4) |
| 73 | Estradiol (E2) |
| 74 | Estriol (E3) |
| 75 | Estrone (E1) |
| 76 | Estrone-sulfate (E1S) |
| 77 | Fibroblast growth factor 2 (FGF2) |
| 78 | Fibroblast growth factor 19 (FGF19) |
| 79 | Fibroblast growth factor 21 (FGF21) |
| 80 | Fibroblast growth factor 23 (FGF23) |
| 81 | Follicle-stimulating hormone (FSH) |
| 82 | Follistatin |
| 83 | Galanin |
| 84 | Gastric inhibitory polypeptide (GIP) |
| 85 | Gastrin |
| 86 | Ghrelin |
| 87 | Glicentin |
| 88 | Glucagon |
| 89 | Glucagon-like peptide-1 (GLP-1) |
| 90 | Glucagon-like peptide-2 (GLP-2) |
| 91 | gonadotropin-releasing hormone (GnRH) |
| 92 | Growth arrest-specific protein 6 |
| 93 | Growth hormone (GH) |
| 94 | Growth hormone-releasing hormone (GHRH) |
| 95 | Growth/differentiation factor 15 (GDF-15) |
| 96 | Guanylin |
| 97 | Hepatocyte growth factor (HGF) |
| 98 | Hepcidin |
| 99 | human placental lactogen |
| 100 | Inhibin A |
| 101 | Inhibin B |
| 102 | Insulin |
| 103 | Insulin-like factor 3 |
| 104 | Insulin-like growth factor I |
| 105 | Insulin-like growth factor II |
| 106 | Insulin-like peptide 5 (INSL5) |
| 107 | Insulin-like peptide 6 (INSL6) |
| 108 | Irisin |
| 109 | Islet amyloid polypeptide (Amylin) |
| 110 | Katacalcin |
| 111 | Kisspeptin |
| 112 | Klotho |
| 113 | Leptin |
| 114 | Leu-enkephalin |
| 115 | Luteinizing hormone (LH) |
| 116 | Melanin concentrating hormone (MCH) |
| 117 | Melanocyte-stimulating hormone (MSH) alpha |
| 118 | Melanocyte-stimulating hormone (MSH) beta |
| 119 | Melanocyte-stimulating hormone (MSH) gamma |
| 120 | Melatonin |
| 121 | Met-Enkephalin |
| 122 | Motilin |
| 123 | Musclin |
| 124 | Myonectin |
| 125 | Myostatin |
| 126 | Natriuretic peptides B |
| 127 | Nesfatin-1 |
| 128 | Neurokinin-A |
| 129 | Neurokinin-B |
| 130 | Neuromedin-B |
| 131 | Neuromedin-C (GRP, (Gastrin releasing peptide, Bombesin-like peptide) |
| 132 | Neuromedin-N |
| 133 | Neuromedin-S |
| 134 | Neuromedin-U |
| 135 | Neuronostatin |
| 136 | Neuropeptide S |
| 137 | Neuropeptide Y (NPY) |
| 138 | Neurotensin |
| 139 | Nociceptin |
| 140 | Norepinephrine (noradrenaline) |
| 141 | Obestatin |
| 142 | Oncostatin-M |
| 143 | Orexin-A |
| 144 | Orexin-B |
| 145 | Osteocalcin |
| 146 | Oxyntomodulin |
| 147 | Oxytocin |
| 148 | Pancreastatin |
| 149 | Pancreatic polypeptide |
| 150 | Parathyroid hormone (PTH) |
| 151 | Parathyroid hormone-related protein |
| 152 | Peptide YY (PYY) |
| 153 | PHM-27 Peptide histidine methionine 27 |
| 154 | Pituitary adenylate cyclase-activating peptide |
| 155 | Placenta growth factor |
| 156 | Pregnenolone |
| 157 | Prengenolone-sulfate |
| 158 | Preptin |
| 159 | Progesterone |
| 160 | Prokineticin 2 |
| 161 | Prolactin |
| 162 | Prolactin-releasing peptide |
| 163 | Promotilin |
| 164 | Relaxins |
| 165 | Renin |
| 166 | Resistin |
| 167 | reverse T3 |
| 168 | Secretin |
| 169 | Serotonin |
| 170 | Somatostatin |
| 171 | Testosterone |
| 172 | Testosterone-sulphate |
| 173 | Thrombopoietin |
| 174 | Thymulin |
| 175 | Thyroid-stimulating hormone (TSH) |
| 176 | Thyrotropin-releasing hormone (TRH) |
| 177 | Tetraiodothyronine (T4) |
| 178 | Triiodothyronine (T3) |
| 179 | Tuberoinfundibular peptide of 39 residues (TIP39) |
| 180 | Urocortin-1 |
| 181 | Urocortin-2 |
| 182 | Urocortin-3 |
| 183 | Urodilatin |
| 184 | Uroguanylin |
| 185 | Urotensin-2 |
| 186 | Vasoactive intestinal peptide (VIP) |
| 187 | Vessel dilator peptide |
| 188 | Visfatin |

### Table B. Suggested compilation of human hormones for clinical endocrinology

This table compiles an extensive list of human hormones, as defined strictly in the context of clinical endocrinology. It includes only bioactive messengers produced that exert effects on distant target cells through ligand-receptor interactions. The selection excludes metabolites, precursors, degradation products, and mediators employed primarily by the immune system. Neuropeptides are included only if they are documented to have endocrine functions. The entries are organized alphabetically. For mean values and reference intervals, refer to S1 Data (sheet: Hormones_comprehensive).

| DHEAS | | | | |
| --- | --- | --- | --- | --- |
| **Population:**  **Sex, ages** | **Group size** | **Measure of central tendency** | **Measure of dispersion** | **Reference** |
| M, 20-30 | 10 | mean 11.5 uM | SEM ± 1.0 uM | (Labrie et al. 1997)[[41]](https://sciwheel.com/work/citation?ids=5267438&pre=&suf=&sa=0) |
| M, 25-29 | 151 | log mean 3320 ng/ml | 90% CI (log space M, 15-39) 1,500-5,500 ng/ml | (Orentreich et al. 1984)[[42]](https://sciwheel.com/work/citation?ids=3115718&pre=&suf=&sa=0) |
| M, 21-30 | 7 | mean 9.6 uM | 95%CI  ± 1.3 uM | (Sulcová et al. 1997)[[43]](https://sciwheel.com/work/citation?ids=7677488&pre=&suf=&sa=0) |
| F, 20-30 | 10 | mean 6.2 uM | SEM of ± 0.5 uM | (Labrie et al. 1997)[[41]](https://sciwheel.com/work/citation?ids=5267438&pre=&suf=&sa=0) |
| F, 25-29 | 63 | log mean 2040 ng/ml | 90% CI (log space F, 15-29) 1,000-5,000 ng/ml | (Orentreich et al. 1984)[[42]](https://sciwheel.com/work/citation?ids=3115718&pre=&suf=&sa=0) |
| F, 21-25 | 9 | mean 8.8 uM | 95%CI ± 0.9 uM | (Sulcová et al. 1997)[[43]](https://sciwheel.com/work/citation?ids=7677488&pre=&suf=&sa=0) |
| Adiponectin | | | | |
| **Population: Sex, ages** | **Group size** | **Measure of central tendency** | **Measure of dispersion** | **Reference** |
| M, 27.5±1.8 | 8 | mean 16.4 ug/ml^*^ | SEM 1.6 ug/ml^*^ | (Ferguson et al. 2004)[[44]](https://sciwheel.com/work/citation?ids=16387774&pre=&suf=&sa=0) |
| M, 35±8 | 17 | mean 9.6 ug/ml | SD 5.7 ug/ml | (Adamczak et al. 2005)[[45]](https://sciwheel.com/work/citation?ids=16387770&pre=&suf=&sa=0) |
| M, 40.3±12.2 | 76 | mean 14.5 ug/ml | SD 4.7 ug/ml | (Fernández-Real et al. 2005)[[46]](https://sciwheel.com/work/citation?ids=16387772&pre=&suf=&sa=0) |
| M, 18-30 | 750 | geomean 8.3 mg/L  median 8.4 mg/L | 25th%-75th% IQR: 6 mg/L | (Steffes et al. 2004)[[34]](https://sciwheel.com/work/citation?ids=16402179&pre=&suf=&sa=0) |
| F, 27.8±2.5 | 8 | mean 19.3 ug/ml^*^ | SEM 2.4 ug/ml^*^ | (Ferguson et al. 2004)[[44]](https://sciwheel.com/work/citation?ids=16387774&pre=&suf=&sa=0) |
| F, 36±9 | 18 | mean 11.7 ug/ml | SD 4.9 ug/ml | (Adamczak et al. 2005)[[45]](https://sciwheel.com/work/citation?ids=16387770&pre=&suf=&sa=0) |
| F, 36.3±10.8 | 40 | mean 17.2 ug/ml | SD 5.2 ug/ml | (Fernández-Real et al. 2005)[[46]](https://sciwheel.com/work/citation?ids=16387772&pre=&suf=&sa=0) |
| F, 18-30 | 865 | geomean 13.6 mg/L  median 14.0 mg/L | 25th%-75th% IQR: 9 mg/L | (Steffes et al. 2004)[[34]](https://sciwheel.com/work/citation?ids=16402179&pre=&suf=&sa=0) |

### Table C. Collated Adiponectin and DHEAS Data from Healthy Young Adult Cohorts

This table summarizes the distribution of DHEAS and adiponectin concentrations in reported cohorts of healthy young adults. Data are presented as reported in the original studies with the following columns: sex and age range, group size, and central tendency measure with corresponding value. Measures of dispersion are reported alongside.

*(*) identifies a corrected discrepancy in unit reporting due to a mismatch between the units used in one cited resource (ng/ml) and the standard units provided by the assay kits (ug/ml).* Underlying data: S1 Data, sheet Hormone_abundance (literature compilation).

| **Hormone** | **individual_CV** | **group_CV** |
| --- | --- | --- |
| 17-OH Progesterone | 0.24 | 0.26 |
| AMH | 0.19 | 0.20 |
| **Adiponectin** | **0.17** | **0.50** |
| Aldosterone | 0.37 | 0.35 |
| Calcitonin | 0.13 | 0.66 |
| Calcitriol | 0.22 | 0.21 |
| Cortisol | 0.16 | 0.30 |
| DHEA | 0.20 | 0.49 |
| **DHEAS** | **0.20** | **0.49** |
| DHT | 0.12 | 0.37 |
| Estradiol, total | 0.15 | 0.18 |
| FGF-23 | 0.14 | 0.23 |
| FSH | 0.10 | 0.38 |
| IGF-1 | 0.09 | 0.27 |
| Insulin | 0.25 | 0.34 |
| LH | 0.25 | 0.21 |
| Osteocalcin | 0.09 | 0.32 |
| PTH | 0.15 | 0.29 |
| Progesterone | 0.19 | 0.40 |
| Prolactin | 0.45 | 0.64 |
| Renin | 0.30 | 0.42 |
| T3, total | 0.06 | 0.12 |
| T4, total | 0.06 | 0.12 |
| TSH | 0.18 | 0.36 |
| Testosterone, total | 0.15 | 0.28 |

### Table D. Collated reference change values (RCVs) for 25 hormones

This table summarizes reference change values compiled from Biological Variation[[19]](https://sciwheel.com/work/citation?ids=18566818&pre=&suf=&sa=0&dbf=0), which quantifies the magnitude of natural fluctuations in hormone levels over time within individuals and across populations. These values were calculated using the formula developed by Fokkema et al[[47]](https://sciwheel.com/work/citation?ids=1363278&pre=&suf=&sa=0&dbf=0). accounting for asymmetrical variation in hormone levels due to their log-normal distribution Underlying data: EFLM Biological Variation Database (URL provided in Methods).
