# Supplementary material for: The total mass, copy number, and distribution of hormones in the human bloodstream
Source: PLoS Biol. 2026 Jun 22;24(6):e3003864. doi: 10.1371/journal.pbio.3003864 (PMC13349298; doi:10.1371/journal.pbio.3003864)
Supplement: Attachment — Submitted filename: rebuttal_letter_PBP.pdf [file pbio.3003864.s006.pdf]

Point-by-Point Response to Reviewers

Manuscript: *Distribution of total hormone mass in human blood*

We thank the Academic Editor and Reviewers for their constructive evaluation. The review process materially improved the manuscript.

The reviewers' comments have been addressed as detailed in the point-by-point responses below.

Prompted by the reviewers' call to move beyond a descriptive census toward quantitative principles and a more rigorously defined scope, we curated and analysed three new datasets — receptor binding affinity, bioavailability fractions, and circulating half-lives — for 56 clinically assayed hormones (new S2 Data). Integration of these data with the original abundance estimates revealed that circulating hormone concentration scales inversely with receptor affinity ($R^2 = 0.31$, $n = 56$, $p < 0.001$) and is structured by chemical class. This analysis yielded two new main figures (Figures 4–5), removal of a previously reported TRH outlier that did not survive stricter validation, and a complete rewrite of the bioavailability section from anecdotal to systematic.

The reviewers also suggested incorporating tissue-specific receptor expression and secretion dynamics. We agree these are important next layers; however, the validation work required for the three new curated datasets consumed the scope of this revision. We frame these extensions explicitly as future directions in the Discussion.

We have also addressed Reviewer 3's concerns regarding scope and framing. Specifically, we removed the term "endocrinome," revised language to reflect the true scope of our dataset (~one-third of known human hormones, selected by availability of standardized clinical assays), toned down claims of "design principles," added external validation using the EFLM Biological Variation Database, and expanded the limitations section.

Sincerely,

Shai Fuchs, MD, PhD (on behalf of the authors)

Reviewer 1

Major Comment 1: Receptor abundance and affinity analysis

> *The interaction between hormones and receptors is the core of endocrine regulation. Extending the quantitative data to abundance and binding affinity of receptors of these hormones and correlating them with hormone levels could be helpful to reveal quantitative principles of signal transduction mediated by these hormones. For instance, the authors have observed a deviation of TRH abundance from the hierarchical structure of the hypothalamic-pituitary axis, as the abundance of TRH is much higher than TSH. The authors suggest that this deviation might reflect additional regulatory roles of TRH, which is possible, but it could also be a consequence of low binding affinity or high abundance of the receptor of TRH. It would also be helpful to determine whether the high-abundance hormones (e.g. DHEA-S) are associated with high receptor density or low binding affinity.*

We curated equilibrium dissociation constants (Kd) for 56 hormones, each assigned a primary cognate receptor (Methods, lines 440-456; S2 Data, sheets "Kd" and "Hormone_affinity_detailed"). Affinities were sourced from IUPHAR/BPS Guide to Pharmacology (GtoPdb) as the primary database, supplemented by an independent AI-assisted literature search with manual verification of every retrieved PMID against its original publication (lines 441-449). We retained one canonical value per hormone–receptor pair, prioritizing human receptor data under near-physiological conditions and applying a hierarchy of Kd > Ki > IC50/EC50 (lines 457-462). Primary receptor assignments were reviewed by the corresponding author (S.F.) and locked prior to statistical analysis (lines 453–454).

In log–log regression, free (receptor-available) concentration predicts receptor Kd (adjusted R^2 = 0.293, n = 56, $p < 0.001$; Results, lines 150–157; Figure S8). Allowing slopes and intercepts to vary by chemical class (amines, peptides, glycoproteins, steroids) substantially improves fit (adjusted $R^2 = 0.384$, lowest AIC = 162.9; $F(6,48) = 5.89$, $p = 5.66 \times 10^{-5}$; Results, lines 158–163; Figure 4C–F; Supplementary Methods, Table Sm1). Eight nested models were compared (Supplementary Methods, lines 794–828; Table Sm1); the class-stratified model was selected on the basis of lowest AIC, highest adjusted R^2, and significant F-test.

Regarding receptor abundance: we agree this is an important next layer. Our original curation plan included tissue-specific receptor expression, with tissue assignment to be determined systematically after completing the affinity, bioavailability, and half-life datasets. However, manual curation proved essential across all three: scripted extraction from established databases (e.g., GtoPdb for Kd) yielded a low survival rate once entries were filtered for the specific scope of our analysis, and AI-assisted literature mining required correction in a significant fraction of cases (Methods, lines 444-449). We expect similar attrition for receptor expression databases (PaxDb, HPA deep proteome), where cross-tissue copy-number estimates would require hormone-by-hormone target-tissue justification and manual verification against the same curation standard. This effort is comparable in scope to the three datasets already added and was not feasible within the revision timeframe. The remaining variance in our models is noted

explicitly as likely reflecting receptor abundance among other unmeasured factors (Discussion, lines 301–304).

Major Comment 2: Mathematical modeling

> *The authors find that for hormones with both inactive reservoir forms and active forms, the abundances of the active forms are much lower than that of the reservoir forms. However, the approach used in this study - direct comparing abundances of the two forms without accounting for the dynamics of interconversion between them - might limit the biological insights one can gain from the data. For instance, why do almost all hormones with both active and inactive forms follow such an abundance pattern? Is the very high abundance of reservoir form indispensable for functions of the hormones, such as robust maintenance of physiological homeostasis and efficient responses to input signals? These important theoretical questions could be addressed by mathematical models simulating the dynamic response of the "reservoir-active" system to external signals.*

The reviewer asked us to move beyond the few descriptive reservoir–active examples in the original submission toward systematic insights, and further suggested adding reaction dynamics as a data layer. We addressed the first request fully. The original descriptive paragraphs and Table 1 have been replaced by a systematic curation of receptor-available fractions and dominant sequestration mechanisms for all 56 hormones (Results, lines 178–197; Figure 5; Figure S9; Methods, lines 471-485; S2 Data, sheet "Bioavailability"). This analysis quantifies the pattern the reviewer identified: sequestration mechanism segregates sharply by chemical class ($\chi^2 = 60.31$, df = 9, $p < 0.001$, Cramér's V = 0.61; lines 185–186), and among sequestered hormones, lower receptor-available fraction associates with longer circulating half-life ($R^2 = 0.40$, $p < 0.001$, n = 32; lines 190–194; Figure 5B). We also curated circulating half-lives for all 56 hormones (Methods, lines 486–503; S2 Data, sheet "Half_life") to provide an initial temporal dimension.

That said, this work remains a steady-state snapshot by design. Dynamic description of the system — from hormone pulsatility to carrier-protein binding kinetics to interconversion rates — is a next-level project that would build on this cross-sectional foundation. We discuss this explicitly as a future direction (Discussion, lines 383-385) and note the temporal limitations of reference-interval-anchored estimates (lines 267–269; 369–372).

Major Comment 3: TRH and peripheral functions

> *The authors hypothesize that the higher level of TRH in the blood compared to other hypothalamic hormones may imply peripheral functions of TRH that have not been fully recognized. However, there is a lack of direct evidence supporting the hypothesis of its peripheral function. Expression of the TRH receptor (TRHR) in peripheral tissues and cell types can be used as an additional source of data to confirm whether TRH may directly affect peripheral tissues. For example, public gene expression databases can be used to retrieve mRNA and protein expression levels of TRHR in peripheral tissues such as thyroid and cardiovascular systems.*

We agree that the original manuscript overreached in inferring peripheral TRH function from abundance data alone. In the revision, we replaced the axis-level hierarchy analysis with a systematic, cross-hormone framework testing the relationship between circulating concentration and receptor binding affinity across 56 hormones (Figure 4). In this broader context, TRH falls within the peptide hormone cluster in the concentration–affinity space (Figure 4E) and does not emerge as an outlier. Its relatively high abundance compared to other hypothalamic hormones remains a noteworthy observation, but we do not believe the data are sufficient to mount a serious challenge to established models of thyroid axis regulation, nor is such a claim within the scope of this work. Within the revised quantitative framework, singling out any one data point around a regression line with substantial residual scatter would not be justified.

Accordingly, we removed the hypothalamic–pituitary axis hierarchy figure and its accompanying discussion. TRH is now treated identically to all other hormones in the dataset. Because the revised manuscript no longer makes a peripheral-function claim for TRH, the receptor-expression analysis the reviewer suggests, while biologically interesting, is no longer needed to support our conclusions.

Major Comment 4: Overstating the take home message of figure 3

> *The take-home-message of Figure 3 is unclear to me. The authors claim that "clear abundance patterns" emerge when comparing the amount and molecular weight of circulating hormones, but Figure 3 contains a mixture of data points with many different colors (secreting organs) and shapes (hormone types), from which I can only see that the abundance and molecular weight of hormones both vary substantially. For "clear patterns" mentioned above, I was expecting quantitative relationships or constraints coupling the variables visualized, which is difficult to see from the figure.*

We agree. The original "clear patterns" language overstated what Figure 3 can show on its own. In the revision, the Results text accompanying Figure 3 now states explicitly that circulating hormone molecule counts "do not manifest any clear pattern" when plotted against molecular weight and tissue origin (lines 124–125). Figure 3 is framed as a descriptive map of the joint distribution of molecular weight and abundance, with iso-mass lines provided as a reading aid. The text then states that this classification does not, on its own, explain hormone abundance (lines 128–129), which serves as the direct motivation for the quantitative analyses in Figures 4 and 5.

Major comment 5: Methodology presentation and details

> *The Methods section is too simplified. For instance, in the subsection "Reference human model and reference conditions", the authors mentioned that a reference human model was used, but it is unclear how this reference model was defined, and what features of this model were used in calculation of the hormone abundances. Was anything other than the body weights of male and*

> *female adults used in the calculation? I suggest the authors provide precise mathematical equations to clarify the calculations they have made.*

We agree that the original Methods section was insufficiently detailed. The revised manuscript substantially expands it — from approximately 67 lines and 6 subsections in the original to approximately 187 lines and 14 subsections, with an additional ~146-line Supplementary Methods section providing formal model equations.

Regarding the reference human: we follow the ICRP Publication 89 convention, a standardized set of anatomical and physiological attributes for a healthy young adult that we have used in prior work (Sender, Fuchs & Milo, *Cell* 2016; Sender, Fuchs & Milo, *PLOS Biol* 2016). The parameters drawn from this model are now stated explicitly (lines 400–408): body mass (73 kg male, 60 kg female), serum volume (3.0 L male, 2.4 L female), reference age (25 years), and standard clinical sampling conditions (healthy, non-pregnant, morning/fasting). For hormones with cyclic physiology, we used nadir-phase values.

Beyond clarifying the reference model, we added six entirely new Methods subsections that were absent from the original:

Hormone–receptor binding affinity (Kd) curation (lines 440–470): two-source search strategy (GtoPdb + AI-assisted PubMed search), manual verification protocol, primary receptor assignment logic, affinity-type hierarchy (Kd → Ki → IC50 with Cheng–Prusoff conversion), and species/assay context documentation.

Bioavailability and receptor-available fraction (lines 471–485): curation of receptor-available fractions, sequestration mechanism annotation, and the operational correction linking clinical assay readouts to receptor-available pools.

Circulating half-life curation (lines 486–502): structured extraction protocol with explicit inclusion/exclusion criteria, terminal β-phase convention, and unit standardization.

Quality control, independence of analysis, and roles (lines 503–511): separation of curation (T.K., S.B., S.F.) from statistical analysis (R.S.), with the finalized data table locked before modeling.

Sensitivity analysis (lines 518-530): DHEAS receptor assignment uncertainty tested across two candidate receptors, showing the concentration–affinity correlation is robust ($R^2 = 0.31 \rightarrow 0.26$; p remains < 0.001).

Biological variation (CVi and CVg) (lines 560–570): EFLM Biological Variation Database query and reporting convention.

Existing subsections were also expanded — for example, the calculation of total circulating mass now explicitly describes conversion to molar abundance and molecule counts and clarifies the use of unbound hormone molecular weight for carrier-bound species (lines 419–430); and the visualization/analysis subsection now details the full statistical testing framework for affinity regression, bioavailability chi-squared tests, and ANOVA (lines 531–547).

Finally, the new Supplementary Methods (lines 769–911) provides all regression model equations in explicit mathematical notation — from univariate through class-stratified and half-life–extended specifications (8 nested models total), with model comparison criteria (F-tests, AIC, BIC), software versions, and implementation details.

Reviewer 2

Comment 1: Brief description of abundant hormones

> *Given the readership of this paper is likely to be broad I think a brief description of the most frequently found hormones might be helpful for interpretation.*

We added a dedicated paragraph in the Discussion (lines 321–341). We defined a "top set" as the union of the five highest-ranking hormones by mass and by molarity in each sex — a stable group of seven: adiponectin, calcidiol, cortisol, DHEAS, IGF-1, IGF-2, and T4. We first orient the reader to the distinct physiological roles these hormones serve - energy metabolism and insulin sensitization, growth and tissue maintenance, the stress response, androgen precursor biology, thyroid regulation, and calcium/bone homeostasis (lines 324–327). We then show that despite this functional diversity, the seven converge on shared circulation-level features: six are extensively carrier-protein bound, three are additionally upstream precursors requiring enzymatic activation, and adiponectin uses oligomeric assembly as a distinct buffering strategy (lines 330–338). This structure addresses the reviewer's request for accessible biological context while advancing the paper's argument - that large circulating pools are maintained by mechanisms decoupling total abundance from immediate receptor exposure, and that abundance reflects buffering architecture rather than physiological importance (lines 339–343).

Comment 2: Circadian rhythms and cortisol

> *There is already some discussion around the fact that hormone levels can fluctuate e.g. in pregnancy or during the menstrual cycle. I think this could be expanded further and discuss that many hormones also follow a circadian rhythm. What approach did you take with cortisol for example (the supplementary file has 3 rows for cortisol)?*

The reviewer raises a point we feel strongly about. The endocrine system is fundamentally dynamic, and a full quantitative account must ultimately capture that. Circulating hormone levels are shaped by multiple superimposed temporal programs spanning orders of magnitude in timescale — from life-stage transitions (most hormones), through seasonal (e.g cortisol) and menstrual cycles, to circadian rhythms, ultradian pulses on the scale of hours, and secretory bursts of minutes or seconds. Layered on top of these are altered physiological states (postprandial, pregnancy, lactation) and pathological ones (disease, pharmacological

intervention). A comprehensive, time-resolved quantitative map of this landscape is, in our view, a long-term goal that will require sustained effort across multiple groups over many years.

The scope decision we made for this study was to begin with a validated steady-state cross-section, precisely because we take granular validation seriously - the multi-step curation and error estimation for even a single time-point inventory proved substantial. The dynamic dimensions will be addressed in follow-up work, and we state this explicitly as a future direction (Discussion, lines 383–388). We believe this cross-sectional foundation is a necessary first layer on which time-resolved analyses can be built.

Within this steady-state framework, we addressed the reviewer's specific concerns as follows. In Methods (lines 403–406), we state that concentrations were interpreted under standard clinical sampling conditions, typically morning/fasting. For cortisol, S1 Data retains three rows to provide full transparency: AM total (5–25 μg/dL), PM total (2–14 μg/dL), and AM free (0.121–1.065 μg/dL). The PM total cortisol value was used for the main analysis, consistent with our approach of using lower-range values as conservative baseline estimates for hormones with pronounced diurnal variation. The AM and free cortisol rows are provided so readers can assess the magnitude of circadian excursion and recalculate if desired.

We also added biological variation data (EFLM Biological Variation Database; Results, lines 260–269; Table S3) reporting within-subject (CVi) and between-subject (CVg) coefficients for 25 hormones, which captures circadian and pulsatile variation as a component of intra-individual dispersion. In the Discussion limitations (lines 369–374), we state explicitly that circadian rhythms, pulsatility, and state dependence are not captured, and argue that because cross-hormone comparisons span multiple orders of magnitude, temporal variation within any single hormone (typically less than 5-fold) is unlikely to alter the qualitative scaling patterns we report.

Comment 3: Rationale for trough-level approach

> *Can the authors please better clarify why they chose to use levels in the cycles where hormones were lowest?*

The choice of nadir-phase values for cyclic hormones (e.g., follicular-phase gonadotropins, PM cortisol) follows from the study's aim: a conservative, reproducible baseline inventory rather than a peak-activity snapshot. Nadir values are the most stable and reproducible sampling context across individuals, minimizing the influence of variable timing within a cycle. They also provide a lower-bound estimate, ensuring that our total mass and molecule-count figures are not inflated by transient peaks. We clarified this rationale in Methods (lines 404–406) and note explicitly that other questions such as peak signaling capacity or integrated 24-hour exposure,would motivate different summary choices.

Comment 4: Pediatric and elderly populations

> *It would strengthen the study to discuss how these findings might apply to or differ in pediatric and elderly populations.*

We agree this context is important. The hormone landscape changes substantially across the lifespan — puberty, pregnancy, menopause, and aging each reshape circulating pools in well-documented ways. Our choice to anchor the analysis in a young-adult reference was deliberate: adult clinical reference ranges are the most standardized and reproducible starting point, and validating even this single cross-section required substantial curation effort. In the Discussion (lines 383–388), we now explicitly list the pediatric and ageing human alongside pregnancy, postprandial states, and disease as priority extensions of the framework. We frame the current dataset as an adult baseline intended to support future age- and state-specific comparisons, rather than as a claim about the endocrine system across all life stages.

Comment 5: Review by a chemical pathologist

> *It would be beneficial for the manuscript to be reviewed by a chemical pathologist with experience in reference range methodology.*

Our methodology was reviewed by Dr. Jonathan Esensten, a clinical pathologist, who evaluated the reference-range derivation approach and found no significant issues requiring correction; he is acknowledged accordingly (line 585). Additionally, co-author Rina Hemi PhD has headed the Endocrine Clinical Laboratory at Sheba Medical Center (a national tertiary referral center) for over two decades and has extensive experience with clinical hormone assays and reference-range methodology. We additionally incorporated EFLM Biological Variation Database entries for 25 hormones (Results, lines 260–269; Table S3), providing an independent, community-curated benchmark for the variability of reference values.

Reviewer 3

We thank Reviewer 3 for the careful and detailed evaluation. We found Reviewer 3's comments highly consequential for improving the manuscript's integrity. The core critique, that the original submission overstated its scope and conflated a quantitative resource with a mechanistic framework, was correct. Several specific gaps the reviewer identified (half-life/clearance, validation breadth, inclusion/exclusion transparency, the distinction between abundance and physiological importance) pointed to the same underlying problem: claims that outran the data.

In revision, we addressed this at two levels. First, we corrected the framing throughout: we removed or replaced language implying comprehensive landscape characterization, explicitly defined the study's scope as clinically assayed circulating hormones in adults, toned down interpretive claims to match what the analyses can support, and stated clearly that abundance is

not a proxy for physiological importance. Second, we added three new curated datasets (receptor binding affinity, bioavailability/sequestration, and circulating half-life), two new main-text figures, and two new supplementary figures that address several of the specific analytical gaps the reviewer raised, particularly around half-life, clearance, and the relationship between circulating pools and receptor engagement. These additions strengthen the resource value the reviewer recognized while keeping inference aligned with the resolution of the data.

Point-by-point responses follow.

General Comment 1: Scope definition and overclaiming

> *The scope of the study is not clearly defined or motivated because the Introduction suggests they will characterize "the entire human hormonal landscape," but they study only those 63 hormones with reference intervals available from Tietz or Mayo. They report 124 other hormones that they justify excluding because they represent negligible mass compared to DHEA-S. They thus do not end up characterizing the entire human hormonal landscape.*

The reviewer is correct that the original framing overreached. The Introduction now describes the study as "the first integrative, quantitative analysis of human hormone abundances, focusing on clinically assayed hormones with established reference ranges" (lines 49–51). The Discussion scope paragraph (lines 352–358) states explicitly that we quantify clinically assayed circulating hormones in adults rather than attempting an exhaustive census of endocrine signaling molecules, and reports the coverage fraction: roughly one third of catalogued hormones, capturing ~95% of total circulating mass. Language implying comprehensive landscape characterization has been removed throughout. See also Specific Comment 1 (removal of "endocrinome" terminology)

General Comment 2: Inclusion/exclusion criteria for precursors and prohormones

> *The Discussion claims that their study presents a "quantitative framework" that provides "key insights" into "organization and regulation." I see a few interesting hypotheses regarding organization and regulation but no clear validated "key insights." I see a valuable and significant quantitative resource that seems to complement other available resources (like Tietz and biologicalvariation.eu) but no clear framework in terms of tools or new principles that could be readily used by others to provide insights.*

We accept this criticism. The original Discussion conflated assembling a quantitative resource with deriving mechanistic principles from it. In the revision, we recalibrated throughout. The Discussion no longer claims "key insights into organization and regulation." Instead, we frame the primary contribution as placing diverse hormone families on a common quantitative scale (lines 271–278) and describe the patterns we observe as reproducible quantitative structure that motivates testable hypotheses, not as validated principles (lines 375–382). The closing passage refers to "regularities and outliers" rather than regulatory insights.

That said, we believe the revision moves the paper beyond resource alone. The three new curated datasets and four new figures (two main, two supplementary) introduce quantitative relationships absent from the original: a concentration-affinity correlation structured by chemical class (adj. $R^2 = 0.384$, $p = 5.66 \times 10^{-5}$; Figure 4), class-segregated sequestration mechanisms ($\chi^2 = 60.31$, $p < 0.001$; Figure 5A), and an inverse association between receptor-available fraction and half-life among sequestered hormones ($R^2 = 0.40$, $p < 0.001$; Figure 5B). These are correlational and partial, as we state explicitly, but they identify structure beyond inventory. We hope the reviewer agrees this represents a meaningful step between "resource" and "framework," even if the latter remains aspirational. See also Specific Comments 3b–c (removal of unsupported pattern claims) and 3f (abundance distinguished from physiological importance).

General Comment 3: Precursors and prohormones

> *Also, they need to explain or provide context for their choices to include some precursor molecules and prohormones and not others. It seems like those choices are made on the basis of whether a clinical assay exists. It doesn't obviously make sense that all biologically-relevant hormones must also be clinically relevant.*

The reviewer is right that the primary inclusion criterion is availability of a clinically validated reference interval, and we now state this transparently (Introduction, lines 49–51; Methods, lines 572-582). This is a pragmatic boundary, not a biological judgment: we do not claim that clinically assayed hormones are the only biologically relevant ones, nor that exclusion implies lack of physiological significance. We chose this cohort because clinical reference intervals provide the most standardized, reproducible, and externally validated concentration data available, which matters when the goal is cross-hormone quantitative comparison with controlled error estimation.

We note that the revision engages directly with the biology of precursor molecules rather than treating them as inert. Our affinity curation reveals that the conventional active/inactive distinction lacks nuance: T4 engages the same thyroid hormone receptors as T3 but with 10–30-fold lower affinity (lines 330–331), calcidiol binds VDR at ~1000-fold lower affinity than calcitriol (line 331–332), and DHEAS, long considered a passive reservoir, has documented receptor engagement at sigma-1 and a recently characterized GPCR (lines 332–334, Methods lines 518-530). These observations are enabled precisely by placing precursors and active forms on the same quantitative scale, which is the approach the reviewer questions.

We agree that extending coverage to biologically relevant hormones lacking clinical assays is an important next step, but note that such hormones are typically less well characterized with respect to circulating ranges, binding parameters, and clearance, making validated quantitative comparison noisier (Discussion, lines 359–362). See also Specific Comment 4a for a detailed discussion of the exclusion categories and the hepcidin example.

General Comment 4: Half-life and clearance

> *The authors also do not discuss hormone half-life or clearance. They mention that some hormones fluctuate but do not provide any detail on fluctuations, which are fundamental to many endocrine functions and would seem very important if the goal is to "characterize the entire human hormonal landscape.*

This gap has been filled. We curated circulating half-lives for hormones from primary literature (Methods, lines 486–502; S2 Data, sheet "Half_life"). Half-life is incorporated in two analyses: as a covariate in the affinity regression models, where it provided no incremental explanatory power beyond concentration (Results, lines 155–157); and in the bioavailability analysis, where receptor-available fraction is inversely associated with half-life among sequestered hormones ($R^2 = 0.40$, $p < 0.001$; Figure 5B, lines 192–194). The Discussion addresses clearance as a class-linked constraint shaping both circulating pool size and receptor-accessible exposure (lines 309–320), and identifies time-resolved secretion and clearance as a priority for future work (lines 383–384). See also Specific Comment 4b.

General Comment 5: Validation and EFLM comparison

> *The use of existing reference intervals from Tietz/Mayo to estimate typical values is clever but potentially superficial. A comprehensive analysis based on one data source, especially one that the authors did not assemble themselves and whose idiosyncrasies are therefore not known to the authors, would benefit from an assessment of potential bias. The authors validate their estimates of total hormonal mass by comparing studies of DHEA-S and adiponectin, but that's only the first step in much more validation that would need to be done given their goal to characterize the entire landscape. The EFLM database provides some overlapping and complementary information.*

We address this fully in Specific Comment 2 below, including our reasoning about the relative scale of inter-laboratory variation versus the cross-hormone spread, the original cohort-level validation (Table 1), and the new EFLM Biological Variation analysis (Table S3).

Specific Comment 1: "Endocrinome" terminology

> *Abstract: "endocrinome" does not seem to be an established term and is not obviously well-defined and seems confusing to use this early. Introducing a new term and saying this study is the "first" to study it is not self-evidently relevant.*

We agree. The term "endocrinome" has been removed from the manuscript entirely. The abstract now describes the work as "the first integrative analysis of circulating human hormones, leveraging clinically validated reference ranges across major endocrine subsystems" (lines 23–25), which defines the scope without introducing non-standard terminology. The "first" claim is retained in this narrower, factual sense: to our knowledge, no prior study has placed this set of clinically assayed hormones on a common quantitative scale with systematic validation.

Specific Comment 2: Limits of Mayo reference intervals; EFLM comparison

> *Methods: What are potential limits and biases of focusing on Mayo reference intervals alone - would comparison with other public resources like EFLM provide some initial guesses?*

This question speaks directly to the precision of the absolute values in our analysis, and we spent considerable time and thought on it. Reference intervals are indeed lab- and platform-specific, with inter-laboratory variation at the same scale or smaller than between-subject biological variation (median $CV_g \approx 0.31$; Table S3). We considered synthesizing intervals across multiple sources, but concluded that this would introduce considerable complexity while adding little analytical power: our quantitative findings emerge from log-log relationships spanning multiple orders of magnitude, where such variation contributes modestly to residual scatter. Moreover, the more fundamental source of uncertainty in this work is the decision to represent a dynamic system as a steady-state cross-section. That choice, not which laboratory generated the reference ranges, is where most of the unknown enters, and we now state this explicitly in the Discussion limitations (lines 369–374).

The original submission already included a direct validation for the two hormones dominating total mass (adiponectin and DHEAS, together >90%): we retrieved all available published data points from independent cohorts and found agreement within 11% of our reference-range-derived estimates (Results, lines 231–239; Table 1). In response to the reviewer's suggestion, we additionally incorporated EFLM Biological Variation Database entries for 25 hormones (Results, lines 260–269; Table S3), providing an independent benchmark for within- and between-subject variability and confirming that population-level dispersion is modest relative to the cross-hormone spread in our analyses.

Specific Comment 3a: One million hormones per cell

> *The finding of about 1M hormones circulating/cell is interesting.*

We thank the reviewer. We agree that this order-of-magnitude ratio is not intuitive, and it illustrates the kind of cross-system perspective that a common quantitative scale can provide. The finding is retained in the revised manuscript (Results, lines 100–101).

Specific Comment 3b+3c: Quantification of "unveiled patterns"

> *b. Comparing abundances in units of mass and mol is interesting but I don't see any "unveil[ed] patterns that potentially correlate with hormone function, glandular origin, and regulatory scheme." Are these potential correlations quantified somewhere?*
> *c. I don't see any "clear abundance patterns" in Figure 3, and I'm not sure what the authors' null hypothesis would be for abundance patterns.*

We thank the reviewer for helping us review and remove insufficiently supported claims such as this one. The reviewer was right on both counts. The original manuscript claimed patterns in Figure 3 that were not quantified and, as the reviewer observed, not visually apparent either. In the revision, we removed this language. The Results text accompanying Figure 3 now states the opposite: circulating hormone molecule counts "do not manifest any clear pattern" when plotted against molecular weight and tissue origin (lines 124–125), and classification by these variables does not, on its own, explain hormone abundance (lines 127–129). Figure 3 is now framed as a descriptive map, and its primary role in the narrative is motivational: because tissue origin and molecular weight fail to account for the eight-order-of-magnitude spread, we ask what other factors might. The quantitative answers come in Figures 4 and 5, where concentration-affinity scaling (adj. $R^2 = 0.384$, $p = 5.66 \times 10^{-5}$), class-segregated sequestration ($\chi^2 = 60.31$, $p < 0.001$), and the half-life/bioavailability relationship ($R^2 = 0.40$, $p < 0.001$) provide the structure the reviewer found missing.

Specific Comment 3d: Figure 4 – HPA bias, ADH, oxytocin

> *Figure 4 shows an interesting pattern, but is that an unbiased assessment of HPA-related molecules, or have the authors limited to those with similar patterns? What about ADH, oxytocin, and others?*

First, on the broader point: we abandoned the attempt to identify abundance patterns associated with secreting glands. The original analysis was already limited to the hypothalamic-pituitary axes, and within those to a subset. While the sequential amplification pattern shown in the old Figure 4 holds for the axes examined, we concluded it is not generalizable to the entire endocrine system and is better explored in a separate, narrower-scope study. The revised manuscript removes that figure entirely and focuses instead on the systematic cross-hormone correlations presented in the new Figures 4 and 5.

To the reviewer's specific examples: ADH (vasopressin) is included in our primary 63-hormone cohort and in the 56-hormone affinity analysis. Oxytocin is not, as it lacks a standardized clinical reference interval in our primary source; it appears in the comprehensive screening list (S1 Data) with negligible contribution to total mass. Importantly, both are posterior pituitary hormones that do not participate in the classical hypothalamic-pituitary-target gland scheme: they are not induced by a hypothalamic releasing factor, nor do they stimulate a downstream endocrine gland. This is the reason they were not included in the original axis hierarchy figure, and while that selection is defensible on physiological grounds, the analysis is no longer part of the revised manuscript for the broader reasons discussed above.

Specific Comment 3e: Serum versus plasma

> *The authors don't discuss the difference between serum and plasma or whether some of the assays whose reference intervals they analyze may actually be plasma measurements. For understanding*

> *physiology, I would think plasma concentrations would be closer to those in vivo and therefore more relevant.*

The reviewer is correct that plasma more closely approximates the in vivo state. In routine clinical practice, however, serum is the standard matrix for the large majority of hormone assays, and reference intervals are reported accordingly. We now address this explicitly. In Methods (lines 425–428), we state that the vast majority of reference intervals used are serum-based, and identify the exceptions: a small number of hormones (ACTH, PTH, and renin in some assays) are preferentially measured in plasma due to stability requirements, and we used the matrix specified by the source for each. In the Discussion limitations (lines 363–365), we acknowledge serum-versus-plasma heterogeneity as one component of measurement matrix variability. For the purposes of this analysis, the distinction is unlikely to alter our conclusions: serum and plasma concentrations for most hormones differ by a small factor (reflecting the removal of clotting factors and fibrinogen), and our cross-hormone comparisons span multiple orders of magnitude.

Specific Comment 3f: Abundance does not equal physiological importance

> *The authors seem to assume that physiologic importance is strongly determined by abundance: "our analysis captures the vast majority of circulating hormone abundance, providing a reliable quantitative framework for understanding endocrine physiology." If their goal is truly to understand endocrine physiology, I don't think that makes sense. It implies that DHEA-S and adiponectin are the most important molecules to study in order to understand endocrine physiology.*

The reviewer is right, and the original phrasing invited exactly this reading. We revised the manuscript to separate these two ideas in multiple places. The sentence the reviewer quoted has been shortened to state only that our analysis "captures the vast majority of circulating hormone abundance" (line 259), without the implication that this equates to understanding physiology. Early in the Results, we now note that "while total mass provides insight into biosynthetic investment, the biological impact of hormones depends on their molecular interactions with receptors" (lines 81–82), redirecting toward the affinity analyses that follow. The Discussion paragraph on the top-set hormones (lines 321–341) shows that high abundance consistently emerges from sequestered, hypoactive, or inactive reservoir forms: six of the seven most abundant hormones are extensively carrier-protein bound, three are upstream precursors requiring enzymatic activation to more potent forms, and the paragraph concludes that "low-abundance hormones such as insulin and GnRH exert outsized physiological effects through high receptor affinity and signal amplification. Abundance therefore reflects circulation-level buffering architecture, not physiological importance" (lines 339–341). Throughout the revision, abundance is treated as a quantitative trait to be explained, not as a ranking of functional significance.

Discussion Comment 4a: Inclusion/exclusion clarity – hepcidin example

> *"By clearly delineating inclusion and exclusion criteria": unless the authors mean simply that they relied on the presence of a reference interval, these criteria are not clear. After a few searches to try to understand why, for instance, hepcidin is not included, it looks like its exclusion criterion is "transduction/structural," but I don't understand what that means.*

The inclusion criterion for the primary 63-hormone cohort is straightforward and now stated explicitly: availability of a clinically validated reference interval for a circulating endocrine hormone in healthy adults (Introduction, lines 49–51; Methods, lines 566–576). The broader catalogue of ~187 hormones was classified by the corresponding author (S.F.) with per-molecule exclusion rationale documented in the supplementary tables (S1 Data, sheets "Uniprot_query" and "HorDB_query"; Supplementary Figures 6–7). The author revised his previously manually curated decisions, this time using an independent LLM query (ChatGPT 5.0, OpenAI) to review existing designations followed by a manual revisit of flagged entries. That query led to changing designations of three entries detailed in the *Additional Corrections* section. We recognize that some boundary cases, such as whether IL-6 is best classified as a hormone or an immune messenger, are genuinely debatable. We consider this transparency a feature, not a limitation: the field would benefit from an explicit, discussable short list of hormones in the endocrine sense, with the inevitably gray borders open for scrutiny.

Regarding the specific example of hepcidin: we believe there may be a misidentification. Hepcidin is marked "Included" in both database queries (UniProt query, row 1221; HorDB query, Hepcidin-25), and appears in the comprehensive hormone list (S1 Data) with an estimated circulating mass of ~65 µg. The "transduction/structural" label the reviewer encountered is applied to neighboring iron-pathway proteins in the UniProt query (erythroferrone, ferroportin), not to hepcidin itself. In the HorDB query, the active isoform Hepcidin-25 is included, while Hepcidin-20, a truncated inactive isoform, is excluded as a "degradation product." Hepcidin is absent from the primary 63-hormone cohort only because it lacks a standardized clinical reference interval in our primary source.

Discussion Comment 4b: Production rates and half-lives

> *There is a statement about negligible "production," but have they inferred or discussed any rates of production or half-lives?*

The original manuscript made a comparison between total circulating hormone mass (~40 mg) and daily whole-body biomass turnover (~100 g) without providing any hormone-specific temporal data. This has been substantially addressed. We curated circulating half-lives for all 56 hormones from primary literature (Methods, lines 486–502; S2 Data, sheet "Half_life"). Half-life enters the analysis in two ways: as a covariate in the affinity regression models, where it provided no incremental explanatory power beyond concentration alone (Results, lines 155–157); and in the bioavailability analysis, where receptor-available fraction is inversely

associated with circulating half-life among sequestered hormones ($R^2 = 0.40$, $p < 0.001$; Figure 5B, lines 192–194).

We retained the original comparison of aggregate hormone mass to whole-body turnover (Discussion, lines 285–286) as a scaling context, but the revised text frames it as motivation for asking what does shape abundance, rather than as a claim about production. We do not calculate hormone-specific production rates explicitly; at steady state these are implicitly constrained by pool size and half-life, but estimating them rigorously would require accounting for distribution volumes and multi-compartment clearance kinetics, which is beyond the scope of this cross-sectional analysis. We note secretion, clearance, and time-resolved dynamics as future directions (Discussion, lines 383–384).

Overall tone

> *[Phrasing, reserved tone overall]*

We have systematically toned down claims throughout the manuscript. Specific changes include: removing “endocrinome”; replacing “entire human hormonal landscape” with scoped language; removing unsupported claims of “key insights” and “design principles”; adding explicit uncertainty language to correlational findings (e.g., “consistent with,” “compatible with”); and adding the “abundance ≠ importance” statement. The revised text frames all findings as correlational observations, not validated principles.

Additional corrections identified by the authors

Hormone list curation: Three entries were removed from the comprehensive hormone list (Table S1, S1 Data): Neurophysin 1 and Neurophysin 2, reclassified as carrier proteins rather than hormones; and Prolactin-releasing peptide, which lacks confirmed human plasma measurement and endocrine function was reclassified as a neuropeptide. Three entries were added: 17-OH pregnenolone sulfate and Dopamine-sulfate (sulfated circulating conjugates identified during the bioavailability analysis) and Asprosin (a recently characterized fasting-induced metabolic hormone). The revised list contains 187 hormones. Changes are color-coded in S1 Data (sheet: Hormones_comprehensive): red for removals, blue for additions and corrected values. These annotations will be resolved for the publication version.

Pregnenolone-sulfate decimal error: A decimal point error in the pregnenolone-sulfate entry was corrected in S1 Data (highlighted in blue), which revised the extended hormone mass estimate from ~1.6 mg to ~2.2 mg (~6% of total). This does not affect any conclusions.

Angiotensin II isoform: We identified and corrected a reference to the less active isoform of Angiotensin II in S1 Data, now correctly referencing the primary bioactive form (Ang II 1–8).

Data file hygiene: Both supplementary data files (S1 Data, S2 Data) have been systematically cleaned: spelling standardized across receptor names, trailing empty rows/columns removed, unit labels verified (half-life units confirmed as seconds throughout), and Readme sheets updated.
