# Supplementary material for: The total mass, copy number, and distribution of hormones in the human bloodstream
Source: PLoS Biol. 2026 Jun 22;24(6):e3003864. doi: 10.1371/journal.pbio.3003864 (PMC13349298; doi:10.1371/journal.pbio.3003864)
Supplement: Attachment — Submitted filename: Response to Reviewers R3.docx [file pbio.3003864.s007.docx]

**Response to Reviewers — R3**

*Manuscript: PBIOLOGY-D-25-00198R2*

We thank the editor and reviewers for their continued engagement with this manuscript. Reviewer 1 reported no further concerns, and Reviewer 3 raised thoughtful general comments and seven specific points, all of which we have considered carefully. Below we respond in detail, indicating where each comment is reflected in the revised text or figures. The locked S1/S2 data, the figures derived from them, and all principal conclusions are unchanged.

# **Reviewer 1**

*“The authors have addressed my previous comments. I have no further concerns.”*

We thank Reviewer 1 for the positive evaluation.

# **Reviewer 3 — general comments**

Reviewer 3’s general comments — about why some hormones sit far from their Kd, why peptide and glycoprotein within-class correlations are weaker than one might naively expect, and the contrast between adiponectin’s circulating prominence and its minimal clinical use — were the most substantive points in the review. We treated them seriously and discussed at length whether to add a new paragraph of interpretation. In the end we concluded that the most honest response is to let the data and the existing framework carry the reviewer’s point, with a single targeted addition where the reviewer identifies a genuinely unaddressed gap.

## **General comment 1 — weaker correlations for peptides and glycoproteins; hormones far from Kd**

*“The modestly significant correlation between hormone amount and binding affinity makes sense and might have been anticipated… maybe the more interesting finding is that the empirical correlations might be weaker (especially for peptides and glycoproteins) than one might have expected. Why would some hormones have typical levels far from their Kd?”*

We agree this is the more informative framing, and we wanted the figure itself to communicate it. Per the reviewer’s related Specific Comment 3, panels C–F of Figure 4 now display per-panel R² and within-class slope p-values. The reader sees immediately that the steroid fit is reasonably strong (R² = 0.398, p = 0.009), the amine fit is borderline (R² = 0.548, p = 0.057, n = 7), and the peptide and glycoprotein fits are weak (R² = 0.089 and 0.176; p = 0.139 and 0.349). The result is now visually self-disclosing — the reviewer’s observation is built directly into the figure, reinforcing the class-linked differences the manuscript text already discusses (below).

Two existing passages in the manuscript carry the reviewer’s point. First, the class-stratification paragraph discusses how class-linked constraints — size, solubility, carrier binding, and clearance — affect both circulating pool size and receptor-accessible exposure. Second, the top-set paragraph concludes that abundance reflects circulation-level buffering architecture rather than physiological importance, and is now further reinforced (see next comment). We have kept the interpretation proportionate to what the cross-sectional dataset can support rather than enumerating candidate mechanisms the data cannot adjudicate.

## **General comment 2 — adiponectin and physiological importance**

*“The centrality of adiponectin in the study based on its total mass contrasts sharply with its apparently minimal clinical relevance… Its greatest significance may be as evidence that the quantity of a hormone is not relevant for assessing its importance.”*

The reviewer is right, and this point deserved direct acknowledgement in the manuscript text. We have added one sentence to the existing top-set paragraph, immediately after the line that frames the seven hormones as “maintained by mechanisms that decouple total abundance from immediate receptor exposure”:

*“Adiponectin is a case in point: total mass is the largest of any circulating hormone, yet it barely figures in routine clinical practice.”*

That sentence sits within the existing paragraph rather than as a stand-alone insertion, and it leads naturally into the existing conclusion that abundance reflects circulation-level buffering architecture, not physiological importance.

## **General comment 3 — Figure 5 patterns and the within-class diversity**

*“Figure 5: Except for glycoproteins, there’s not much of a consistent pattern based on type.”*

We accept this critique. The class-by-class breakdown shows that the cleanest segregation is between glycoproteins (7/7 with no sequestration) and steroids (16/16 with carrier binding), while amines and peptides display real within-class diversity. We have removed the strength qualifier from the Figure 5 caption, which now reads “associates with sequestration mechanism” rather than “associates strongly with sequestration mechanism.” The chi-squared result remains highly significant statistically, but the unqualified wording no longer overstates what the panel shows visually.

# **Reviewer 3 — specific comments**

## **Specific Comment 1 — “first integrative analysis”**

*“Abstract and line 48: ‘the first integrative analysis of circulating human hormones….’ Is this study really the first that any reasonable person might call an ‘integrative analysis of circulating hormones’? Based on what I have read, it’s fair to say it’s one of the largest.”*

Agreed and corrected. “The first” has been replaced by “a comprehensive,” in both the Abstract and the Introduction.

## **Specific Comment 2 — adiponectin and “routinely assayed”**

*“Line 59: The irony is that adiponectin is not ‘routinely assayed’ because it has almost no clinical utility that I am aware of.”*

Acknowledged with thanks. The relevant sentence in the Introduction now reads: “63 hormones, mostly routinely assayed in medical laboratories (with adiponectin and a minority of others primarily measured in research and specialty contexts), spanning the endocrine glands and hormone-secreting tissues.” This both flags adiponectin specifically — picking up the reviewer’s exact example — and accurately describes the cohort.

## **Specific Comment 3 — Figure 4 panels and per-class R²**

*“Figure 4: is there any correlation in panels E and F? I think R² should be provided in all panels.”*

Figure 4 has been updated. Panels C–F now display per-panel R² right-aligned with the existing y=… equation in each panel, mirroring the layout used in panels A and B. As the reviewer anticipated, the within-class fits for peptides (panel E) and glycoproteins (panel F) are weak, which the new annotations now make visually explicit:

| **Panel** | **Class** | **n** | **R²** | **p (slope)** |
| --- | --- | --- | --- | --- |
| C | Steroids | 16 | 0.398 | 0.009 |
| D | Amines | 7 | 0.548 | 0.057 |
| E | Peptides | 26 | 0.089 | 0.139 |
| F | Glycoproteins | 7 | 0.176 | 0.349 |

The original figure design is otherwise untouched. As discussed in our response to General Comment 1, the now-visible per-panel statistics are how we have chosen to communicate the variable within-class fit strength that the reviewer’s general comments highlight.

## **Specific Comment 4 — Figure 5 and class patterns**

Addressed under General Comment 3 above. The Figure 5 caption now reads “associates” without a strength qualifier (previously “associates strongly”), and the accompanying Results paragraph explicitly notes that glycoproteins (7/7) and steroids (16/16) show the cleanest class segregation while amines and peptides display within-class diversity.

## **Specific Comment 5 — tissue-mass hypothesis**

*“Discussion (275-286): this examination of the role of tissue mass and the plausible related hypothesis is useful.”*

We thank the reviewer for this affirmation; the passage has been retained unchanged.

## **Specific Comment 6 — “self-reported” race and ethnicity**

*“Discussion (367): ‘self-reported’ race and ethnicity because there is no other kind”*

Acknowledged and corrected. The qualifier “self-reported ” has been inserted before both “African-American” and “Caucasian” in the relevant Discussion sentence.

## **Specific Comment 7 — “reference interval” vs “reference range”**

*“Throughout: According to Fraser and other laboratory medicine thought leaders, ‘reference interval’ is technically more accurate than ‘reference range’.”*

We have standardized on “reference interval” throughout the main manuscript and the supplementary materials. The single retained instance of “reference ranges” is in the historical sentence in the Introduction (“Clinical reference ranges, first introduced as a concept in the 1960s…”), where the phrase names the original concept.

# **Editor’s formal requirements**

## **Data policy — figure legends**

Every figure legend (main and supplementary) now includes a one-line pointer to the underlying data location: the appropriate S1/S2 sheet, and the Zenodo archive for analysis code. The five Voronoi-based figures (main Figures 1 and 2; Supplementary Figures S1–S3) additionally cite the Proteomaps tool used to generate them (http://bionic-vis.biologie.uni-greifswald.de/).

## **Code policy — Zenodo deposition**

The analysis code that produced Figures 3–5 and Figures S5, S8, S9 is available at https://gitlab.com/milo-lab-public/hormones_distribution (live repository, tagged release v1.0.0) and archived on Zenodo: https://doi.org/10.5281/zenodo.20110628. The Methods sentence on code availability and the Data Accessibility Statement have been updated accordingly.

## **Reference-list audit**

We conducted a final audit of the reference list per the editor’s request, including a retraction-status check via PubMed’s “Retracted Publication” and “Published Erratum” filters for all 47 numbered references and for all 254 supporting PMIDs cited in S1 and S2 Data. No retracted citations or errata were identified. Reference 5, originally cited as a bioRxiv preprint (Kaur et al., Hmrbase2), has since been published in Hormones (Athens); we have updated the citation to the peer-reviewed version: Kaur D, Arora A, Patiyal S, Raghava GPS. Hmrbase2: a comprehensive database of hormones and their receptors. Hormones (Athens). 2023;22(3):359–366. doi:10.1007/s42000-023-00455-5; PMID 37291365. No other reference changes were made.

# **Additional consistency reconciliations identified during final audit**

During final preparation we audited every reportable statistic against the locked S1/S2 data tables. The audit identified a small number of narrative summaries that reflected an earlier curation snapshot rather than the final, locked tables. The locked S1/S2 data, the figures derived from them, the regression statistics in the Figure 4 caption, and all principal conclusions are unchanged. The corrections, all visible in the track-changes manuscript, are listed below for completeness:

| **Item** | **Original** | **Corrected** |
| --- | --- | --- |
| Per-class hormone counts (Supplementary Methods) | Steroid 22, Peptide 19, Glycoprotein 8, Amine 7 | Steroid 16, Peptide 26, Glycoprotein 7, Amine 7 |
| Class-stratified F-statistic df label (Results) | F(6,48) = 5.89, p = 5.66×10⁻⁵ | F(7,48) = 5.89, p = 5.7×10⁻⁵ (matches Fig 4 caption) |
| Discussion p-value (free vs total molarity) | p = 7.2×10⁻⁶ | p = 9.9×10⁻⁶ (matches Fig 4A caption) |
| Glycoprotein sequestration count (Results, Fig 5) | 6/6 | 7/7 (matches Fig 4/5 subset, n=56) |
| Table S3 / EFLM example hormones (Results) | free T4 (CVg ≈ 0.08, CVi ≈ 0.048); free testosterone (CVg ≈ 0.68, CVi ≈ 0.55) | T3 total (CVg ≈ 0.12, CVi ≈ 0.06); prolactin (CVg ≈ 0.64, CVi ≈ 0.45) — both rows actually present in Table S3 |
| Adiponectin %-of-mass single number | roughly 75% in both sexes | split per sex: ≈74% male, ≈83% female |
| UniProt “Included” count (Fig S6) | 69 (3%) | 68 (3%) — matches actual count in S1 Data after stray formula cell relabelled |
| “Additional 127 endocrine molecules” | 127 | 124 (= 187 comprehensive list − 63 primary cohort) |
| Stray Excel cell in S1 → Uniprot_query | row labelled “recept+A2+B1412:B1414” | relabelled to “receptor” |
| Median CVg | ≈0.31 | ≈0.32 |
