# Supplementary material for: The total mass, copy number, and distribution of hormones in the human bloodstream
Source: PLoS Biol. 2026 Jun 22;24(6):e3003864. doi: 10.1371/journal.pbio.3003864 (PMC13349298; doi:10.1371/journal.pbio.3003864)
Supplement: Peer review history

# Editor report: Decision Letter 0

**DOI:** 10.1371/journal.pbio.3003864.r001

**Authors:** Ines Alvarez-Garcia

5 Feb 2025

Dear Dr Fuchs,

Thank you for submitting your manuscript entitled "The total mass, copy number, and distribution of hormones in the human bloodstream" for consideration as a Research Article by PLOS Biology. I am writing on behalf of my colleague, Dr. Ines Alvarez-Garcia who is the handling editor for your paper, but who is out of the office at the moment. I am contacting you to let you know that your manuscript has now been evaluated by the PLOS Biology editorial staff as well as by an academic editor with relevant expertise and we would like to send your submission out for external peer review.

Please accept our apologies for the time it has taken to send you this initial decision. We had wished to discuss your paper with an expert in this field, and it took us a bit longer than normal to find someone who was available to provide advice.

**IMPORTANT: Please note, before we can send your manuscript to reviewers, we need you to complete your submission by providing the metadata that is required for full assessment. To this end, please login to Editorial Manager where you will find the paper in the 'Submissions Needing Revisions' folder on your homepage. Please click 'Revise Submission' from the Action Links and complete all additional questions in the submission questionnaire.

Once your full submission is complete, your paper will undergo a series of checks in preparation for peer review. After your manuscript has passed the checks it will be sent out for review. To provide the metadata for your submission, please Login to Editorial Manager (https://www.editorialmanager.com/pbiology) within two working days, i.e. by Feb 07 2025 11:59PM.

If your manuscript has been previously peer-reviewed at another journal, PLOS Biology is willing to work with those reviews in order to avoid re-starting the process. Submission of the previous reviews is entirely optional and our ability to use them effectively will depend on the willingness of the previous journal to confirm the content of the reports and share the reviewer identities. Please note that we reserve the right to invite additional reviewers if we consider that additional/independent reviewers are needed, although we aim to avoid this as far as possible. In our experience, working with previous reviews does save time.

If you would like us to consider previous reviewer reports, please edit your cover letter to let us know and include the name of the journal where the work was previously considered and the manuscript ID it was given. In addition, please upload a response to the reviews as a 'Prior Peer Review' file type, which should include the reports in full and a point-by-point reply detailing how you have or plan to address the reviewers' concerns.

During the process of completing your manuscript submission, you will be invited to opt-in to posting your pre-review manuscript as a bioRxiv preprint. Visit http://journals.plos.org/plosbiology/s/preprints for full details. If you consent to posting your current manuscript as a preprint, please upload a single Preprint PDF.

Feel free to email us at plosbiology@plos.org if you have any queries relating to your submission.

Kind regards,

Luke

Lucas Smith, PhD

Senior Editor

PLOS Biology

lsmith@plos.org

on behalf of

Ines Alvarez-Garcia, PhD

Senior Editor

PLOS Biology

ialvarez-garcia@plos.org

# Peer review: Decision Letter 1

**DOI:** 10.1371/journal.pbio.3003864.r002

**Authors:** Ines Alvarez-Garcia, Ines Alvarez-Garcia

27 May 2025

Dear Dr Fuchs,

Thank you for your patience while your manuscript entitled "The total mass, copy number, and distribution of hormones in the human bloodstream" was peer-reviewed at PLOS Biology. Please accept also again my sincere apologies for the time it has taken us to provide you with a decision. The manuscript has now been evaluated by the PLOS Biology editors, an Academic Editor with relevant expertise, and by three independent reviewers.

The reviews are attached below. As you will see, the reviewers find the manuscript interesting and worth pursuing for publication, but they also raise several issues that would need to be addressed. Reviewer 1 thinks that the quantitative data to abundance and binding affinity of receptors of these hormones should be extended and correlated with hormone levels to identify quantitative principles of signal transduction mediated by these hormones. In addition, the reviewer mentions that some of the theoretical questions could be addressed using mathematical models simulating the dynamic response of the ‘reservoir-active’ system to external signals, and makes some suggestions to confirm some of the findings and improve the methods. Reviewer 2 thinks that a brief description of the most frequently found hormones should be provided, and the discussion expanded to add how many hormones follow a circadian rhythm and what approach was taken with cortisol. Reviewer 3 mentions that the scope of the study should be clearly defined and that the claims should be toned down because the entire hormonal landscape is not characterised. This reviewer also notes the need to explain or provide context for the choices to include some precursor molecules and prohormones and not others, discuss hormone half-life or clearance and validate better the total hormonal mass estimates.

In light of the reviews, which you will find at the end of this email, we would like to invite you to revise the work to thoroughly address the reviewers' reports. Note that after discussing the reviews with the Academic Editor, we do not think that Reviewer 1's request of addressing some of the theoretical questions using mathematical models is required for publication.

Given the extent of revision needed, we cannot make a decision about publication until we have seen the revised manuscript and your response to the reviewers' comments. Your revised manuscript is likely to be sent for further evaluation by all or a subset of the reviewers.

In addition to these revisions, you will need to complete some formatting changes, which you will receive in a follow up email. A member of our team will be in touch with a set of requests shortly.

We expect to receive your revised manuscript within 3 months. Please email us (plosbiology@plos.org) if you have any questions or concerns, or would like to request an extension.

At this stage, your manuscript remains formally under active consideration at our journal; please notify us by email if you do not intend to submit a revision so that we may withdraw it.

**IMPORTANT - SUBMITTING YOUR REVISION**

Your revisions should address the specific points made by each reviewer. Please submit the following files along with your revised manuscript:

1. A 'Response to Reviewers' file - this should detail your responses to the editorial requests, present a point-by-point response to all of the reviewers' comments, and indicate the changes made to the manuscript.

*NOTE: In your point-by-point response to the reviewers, please provide the full context of each review. Do not selectively quote paragraphs or sentences to reply to. The entire set of reviewer comments should be present in full and each specific point should be responded to individually, point by point.

You should also cite any additional relevant literature that has been published since the original submission and mention any additional citations in your response.

2. In addition to a clean copy of the manuscript, please also upload a 'track-changes' version of your manuscript that specifies the edits made. This should be uploaded as a "Revised Article with Changes Highlighted" file type.

3. Resubmission Checklist

When you are ready to resubmit your revised manuscript, please refer to this resubmission checklist: https://plos.io/Biology_Checklist

To submit a revised version of your manuscript, please go to https://www.editorialmanager.com/pbiology/ and log in as an Author. Click the link labelled 'Submissions Needing Revision' where you will find your submission record.

Please make sure to read the following important policies and guidelines while preparing your revision and fulfil the editorial requests:

a) *PLOS Data Policy*

Please note that as a condition of publication PLOS' data policy (http://journals.plos.org/plosbiology/s/data-availability) requires that you make available all data used to draw the conclusions arrived at in your manuscript. If you have not already done so, you must include any data used in your manuscript either in appropriate repositories, within the body of the manuscript, or as supporting information (N.B. this includes any numerical values that were used to generate graphs, histograms etc.). Please also indicate in each figure legend where the data can be found. For an example see here: http://www.plosbiology.org/article/info%3Adoi%2F10.1371%2Fjournal.pbio.1001908#s5

b) *Published Peer Review*

Please note while forming your response, if your article is accepted, you may have the opportunity to make the peer review history publicly available. The record will include editor decision letters (with reviews) and your responses to reviewer comments. If eligible, we will contact you to opt in or out. Please see here for more details:

https://blogs.plos.org/plos/2019/05/plos-journals-now-open-for-published-peer-review/

c) *Blot and Gel Data Policy*

Please provide the original, uncropped and minimally adjusted images supporting all blot and gel results reported in an article's figures or Supporting Information files. We will require these files before a manuscript can be accepted so please prepare them now, if you have not already uploaded them. Please carefully read our guidelines for how to prepare and upload this data: https://journals.plos.org/plosbiology/s/figures#loc-blot-and-gel-reporting-requirements

d) *Protocols deposition*

To enhance the reproducibility of your results, we recommend that if applicable you deposit your laboratory protocols in protocols.io, where a protocol can be assigned its own identifier (DOI) such that it can be cited independently in the future. For instructions see: https://journals.plos.org/plosbiology/s/submission-guidelines#loc-materials-and-methods

Thank you again for your submission to our journal. We hope that our editorial process has been constructive thus far, and we welcome your feedback at any time. Please don't hesitate to contact us if you have any questions or comments.

Sincerely,

Ines

--

Ines Alvarez-Garcia, PhD

Senior Editor

PLOS Biology

ialvarez-garcia@plos.org

------------------------------------

Reviewers' comments

Rev. 1:

In the manuscript 'The total mass, copy number, and distribution of hormones in the human bloodstream', Sender and colleagues systematically integrated clinical reference ranges and public datasets to quantify the distribution of total mass and copy number of hormones in the human circulation system. Their analyses lead to three main conclusions: (1) Adiponectin and DHEA-S dominate the total abundance of human hormones; (2) abundances of hormones follow the hierarchical structure of the hypothalamic-pituitary axe, except for TRH; (3) active forms of hormones are lower in abundance compared to the reservoir forms.

The manuscript is well-written, and the data is open and transparent. I agree with the authors that absolute quantifications are crucial to gain theoretical insights into how biological processes operate, therefore I appreciate the authors' effort in doing so. However, I feel that there is still space to further improve this work from a systems biology perspective (e.g. with mathematical modeling to interpret the rules underlying the hormone abundance data and yield clearly defined theories), as its current form appears to simply list the numbers without converging to a theory about design principles of the endocrine system as they tried to claim in the abstract.

Major:

1. The interaction between hormones and receptors is the core of endocrine regulation. Extending the quantitative data to abundance and binding affinity of receptors of these hormones and correlating them with hormone levels could be helpful to reveal quantitative principles of signal transduction mediated by these hormones. For instance, the authors have observed a deviation of TRH abundance from the hierarchical structure of the hypothalamic-pituitary axis, as the abundance of TRH is much higher than TSH. The authors suggest that this deviation might reflect additional regulatory roles of TRH, which is possible, but it could also be a consequence of low binding affinity or high abundance of the receptor of TRH. It would also be helpful to determine whether the high-abundance hormones (e.g. DHEA-S) are associated with high receptor density or low binding affinity.

2. The authors find that for hormones with both inactive reservoir forms and active forms, the abundances of the active forms are much lower than that of the reservoir forms. However, the approach used in this study - direct comparing abundances of the two forms without accounting for the dynamics of interconversion between them - might limit the biological insights one can gain from the data. For instance, why do almost all hormones with both active and inactive forms follow such an abundance pattern? Is the very high abundance of reservoir form indispensable for functions of the hormones, such as robust maintenance of physiological homeostasis and efficient responses to input signals? These important theoretical questions could be addressed by mathematical models simulating the dynamic response of the "reservoir-active" system to external signals.

3. The authors hypothesize that the higher level of TRH in the blood compared to other hypothalamic hormones may imply peripheral functions of TRH that have not been fully recognized. However, there is a lack of direct evidence supporting the hypothesis of its peripheral function. Expression of the TRH receptor (TRHR) in peripheral tissues and cell types can be used as an additional source of data to confirm whether TRH may directly affect peripheral tissues. For example, public gene expression databases can be used to retrieve mRNA and protein expression levels of TRHR in peripheral tissues such as thyroid and cardiovascular systems.

4. The take-home-message of Figure 3 is unclear to me. The authors claim that "clear abundance patterns" emerge when comparing the amount and molecular weight of circulating hormones, but Figure 3 contains a mixture of data points with many different colors (secreting organs) and shapes (hormone types), from which I can only see that the abundance and molecular weight of hormones both vary substantially. For "clear patterns" mentioned above, I was expecting quantitative relationships or constraints coupling the variables visualized, which is difficult to see from the figure.

5. The Methods section is too simplified. For instance, in the subsection "Reference human model and reference conditions", the authors mentioned that a reference human model was used, but it is unclear how this reference model was defined, and what features of this model were used in calculation of the hormone abundances. Was anything other than the body weights of male and female adults used in the calculation? I suggest the authors provide precise mathematical equations to clarify the calculations they have made.

Rev. 2:

This is an interesting and topical paper, which provides an alternative perspective on the homeostasis and distribution of hormones in the body. My congratulations to the authors on their novel work.

My comments are more around the discussion and the need to provide some extra context and emphasise some caveats further.

Given the readership of this paper is likely to be broad I think a brief description of the most frequently found hormones might be helpful for interpretation.

There is already some discussion around the fact that hormone levels can fluctuate e.g. in pregnancy or during the menstrual cycle. I think this could be expanded further and discuss that many hormones also follow a circadian rhythm. What approach did you take with cortisol for example (the supplementary file has 3 rows for cortisol)?

Can the authors please better clarify why they chose to use levels in the cycles where hormones were lowest?

At the moment the data refers to healthy young adults (25 years old). Can the authors please include some statements about paediatric and elderly populations?

I am not a chemical pathologist but I think a review by one would be warranted prior to publication.

Rev. 3:

General comments:

The authors focus on a scientifically and clinically important and ambitious topic - what are the system-level organizational and functional properties of the human endocrine system. They provide an impressively detailed inventory of dozens of molecules and perform some straightforward analysis in an effort to identify patterns that might yield mechanistic insights. They have organized a substantial amount of information, particularly in the supplement, and this material would be a valuable resource to the biomedical community.

The Discussion claims that their study presents a "quantitative framework" that provides "key insights" into "organization and regulation." I see a few interesting hypotheses regarding organization and regulation but no clear validated "key insights." I see a valuable and significant quantitative resource that seems to complement other available resources (like Tietz and biologicalvariation.eu) but no clear framework in terms of tools or new principles that could be readily used by others to provide insights.

The scope of the study is not clearly defined or motivated because the Introduction suggests they will characterize "the entire human hormonal landscape," but they study only those 63 hormones with reference intervals available from Tietz or Mayo. They report 124 other hormones that they justify excluding because they represent negligible mass compared to DHEA-S. They thus do not end up characterizing the entire human hormonal landscape.

Also, they need to explain or provide context for their choices to include some precursor molecules and prohormones and not others. It seems like those choices are made on the basis of whether a clinical assay exists. It doesn't obviously make sense that all biologically-relevant hormones must also be clinically relevant.

The authors also do not discuss hormone half-life or clearance. They mention that some hormones fluctuate but do not provide any detail on fluctuations, which are fundamental to many endocrine functions and would seem very important if the goal is to "characterize the entire human hormonal landscape."

The use of existing reference intervals from Tietz/Mayo to estimate typical values is clever but potentially superficial. A comprehensive analysis based on one data source, especially one that the authors did not assemble themselves and whose idiosyncrasies are therefore not known to the authors, would benefit from an assessment of potential bias. The authors validate their estimates of total hormonal mass by comparing studies of DHEA-S and adiponectin, but that's only the first step in much more validation that would need to be done given their goal to characterize the entire landscape. The EFLM database provides some overlapping and complementary information (eg., https://biologicalvariation.eu/search?query=dheas, https://academic.oup.com/clinchem/article/69/1/100/6826991).

Specific comments

1. Abstract: "endocrinome" does not seem to be an established term and is not obviously well-defined and seems confusing to use this early. Introducing a new term and saying this study is the "first" to study it is not self-evidently relevant.

2. Methods: What are potential limits and biases of focusing on Mayo reference intervals alone - would comparison with other public resources like EFLM provide some initial guesses?

3. Results

a. The finding of about 1M hormones circulating/cell is interesting.

b. Comparing abundances in units of mass and mol is interesting but I don't see any "unveil[ed] patterns that potentially correlate with hormone function, glandular origin, and regulatory scheme." Are these potential correlations quantified somewhere?

c. I don't see any "clear abundance patterns" in Figure 3, and I'm not sure what the authors' null hypothesis would be for abundance patterns.

d. Figure 4 shows an interesting pattern, but is that an unbiased assessment of HPA-related molecules, or have the authors limited to those with similar patterns? What about ADH, oxytocin, and others?

e. The authors don't discuss the difference between serum and plasma or whether some of the assays whose reference intervals they analyze may actually be plasma measurements. For understanding physiology, I would think plasma concentrations would be closer to those in vivo and therefore more relevant.

f. The authors seem to assume that physiologic importance is strongly determined by abundance: "our analysis captures the vast majority of circulating hormone abundance, providing a reliable quantitative framework for understanding endocrine physiology." If their goal is truly to understand endocrine physiology, I don't think that makes sense. It implies that DHEA-S and adiponectin are the most important molecules to study in order to understand endocrine physiology.

4. Discussion:

a. "By clearly delineating inclusion and exclusion criteria": unless the authors mean simply that they relied on the presence of a reference interval, these criteria are not clear. After a few searches to try to understand why, for instance, hepcidin is not included, it looks like its exclusion criterion is "transduction/structural," but I don't understand what that means.

b. There is a statement about negligible "production," but have they inferred or discussed any rates of production or half-lives?

# Author comment: Author response to Decision Letter 2

**DOI:** 10.1371/journal.pbio.3003864.r003

4 Mar 2026

Attachment Submitted filename: rebuttal_letter_PBP.pdf

# Peer review: Decision Letter 2

**DOI:** 10.1371/journal.pbio.3003864.r004

**Authors:** Ines Alvarez-Garcia, Ines Alvarez-Garcia, Ines Alvarez-Garcia

8 May 2026

Dear Dr Fuchs,

Thank you for your patience while we considered your revised manuscript entitled "The total mass, copy number, and distribution of hormones in the human bloodstream" for publication as a Research Article at PLOS Biology. This revised version of your manuscript has been evaluated by the PLOS Biology editors, the Academic Editor and two of the original reviewers.

Based on the reviews (attached below), we are likely to accept this manuscript for publication, provided you satisfactorily address the remaining points raised by Reviewer 3. We do agree with the reviewer that "the first integrative analysis of circulating human hormones…” should be changed to “an integrative (or, a comprehensive, if preferred) analysis of circulating human hormones." Please also make sure to address the data and other policy-related requests stated below my signature.

As you address these items, please take this last chance to review your reference list to ensure that it is complete and correct. If you have cited papers that have been retracted, please include the rationale for doing so in the manuscript text, or remove these references and replace them with relevant current references. Any changes to the reference list should be mentioned in the cover letter that accompanies your revised manuscript.

In addition to these revisions, you may need to complete some formatting changes, which you will receive in a follow up email. A member of our team will be in touch with a set of requests shortly. If you do not receive a separate email within a few days, please assume that checks have been completed, and no additional changes are required.

We expect to receive your revised manuscript within two weeks.

To submit your revision, please go to https://www.editorialmanager.com/pbiology/ and log in as an Author. Click the link labelled 'Submissions Needing Revision' to find your submission record. Your revised submission must include the following:

- a cover letter that should detail your responses to any editorial requests, if applicable, and whether changes have been made to the reference list

- a Response to Reviewers file that provides a detailed response to the reviewers' comments (if applicable, if not applicable please do not delete your existing 'Response to Reviewers' file.)

- a track-changes file indicating any changes that you have made to the manuscript.

NOTE: If Supporting Information files are included with your article, note that these are not copyedited and will be published as they are submitted. Please ensure that these files are legible and of high quality (at least 300 dpi) in an easily accessible file format. For this reason, please be aware that any references listed in an SI file will not be indexed. For more information, see our Supporting Information guidelines:

https://journals.plos.org/plosbiology/s/supporting-information

*Published Peer Review History*

Please note that you may have the opportunity to make the peer review history publicly available. The record will include editor decision letters (with reviews) and your responses to reviewer comments. If eligible, we will contact you to opt in or out. Please see here for more details:

https://plos.org/published-peer-review-history/

*Press*

Should you, your institution's press office or the journal office choose to press release your paper, please ensure you have opted out of Early Article Posting on the submission form. We ask that you notify us as soon as possible if you or your institution is planning to press release the article.

*Protocols deposition*

To enhance the reproducibility of your results, we recommend that if applicable you deposit your laboratory protocols in protocols.io, where a protocol can be assigned its own identifier (DOI) such that it can be cited independently in the future. Additionally, PLOS ONE offers an option for publishing peer-reviewed Lab Protocol articles, which describe protocols hosted on protocols.io. Read more information on sharing protocols at https://plos.org/protocols?utm_medium=editorial-email&utm_source=authorletters&utm_campaign=protocols

Please do not hesitate to contact me should you have any questions.

Sincerely,

Ines

--

Ines Alvarez-Garcia, PhD

Senior Editor

PLOS Biology

ialvarez-garcia@plos.org

------------------------------------------------------------------------

DATA POLICY:

Thank you for providing the data underlying all the graphs shown in the figures. However, you should indicate clearly in each corresponding figure legend where the data can be found. You can include the link to Zenodo (see our Code policy below) to access the code to generate it.

------------------------------------------------------------------------

CODE POLICY

Per journal policy, if you have generated any custom code during the course of this investigation, please make it available without restrictions. Please ensure that the code is sufficiently well documented and reusable, and that your Data Statement in the Editorial Manager submission system accurately describes where your code can be found. More information on our Code Policy, what and how to share can be found here: https://journals.plos.org/plosbiology/s/code-availability

Please note that we cannot accept sole deposition of code in GitHub, as this could be changed after publication. However, you can archive this version of your publicly available GitHub code to Zenodo. Once you do this, it will generate a DOI number, which you will need to provide in the Data Accessibility Statement (you are welcome to also provide the GitHub access information). See the process for doing this here: https://docs.github.com/en/repositories/archiving-a-github-repository/referencing-and-citing-content

------------------------------------------------------------------------

Reviewers' comments

Rev. 1:

The authors have addressed my previous comments. I have no further concerns.

Rev. 3:

General comments

The authors responded thoughtfully and thoroughly to reviewer comments and substantially revised the paper, including new data, analysis, and figures. It is clearly improved.

The modestly significant correlation between hormone amount and binding affinity makes sense and might have been anticipated, but I am not aware of clear evidence-based demonstration of this relationship, especially not across as many hormones as is demonstrated in this manuscript. If this relationship is what might be naively expected because it would enhance physiological responsiveness, maybe the more interesting finding is that the empirical correlations might be weaker (especially for peptides and glycoproteins) than one might have expected. Why would some hormones have typical levels far from their Kd?

Similarly, is the correlation between receptor-available fraction and half-life what would be naively expected? If so, then maybe what's interesting is that there is, for example, a surprisingly large range of receptor-available fractions (100x) for half-lives between 0.1 and 1 hours.

The centrality of adiponectin in the study based on its total mass contrasts sharply with its apparently minimal clinical relevance and seems to distract the reader from the prior two interesting observations. I cannot remember ever seeing an adiponectin level reported in a patient medical record and am not aware that measurement of adiponectin is recommended in a single clinical practice guideline for screening, or disease progression, or treatment response monitoring. Its greatest significance may be as evidence that the quantity of a hormone is not relevant for assessing its importance. In lines 338-340 of the Discussion, the authors rightly note that abundance does not reflect physiological importance, and if they could come up with a better metric of physiologic importance, a future study focusing on the most physiologically important hormones might yield greater insight.

Specific comments

Abstract and line 48: "the first integrative analysis of circulating human hormones…." Is this study really the first that any reasonable person might call an "integrative analysis of circulating hormones"? Based on what I have read, it's fair to say it's one of the largest.

Line 59: The irony is that adiponectin is not "routinely assayed" because it has almost no clinical utility that I am aware of.

Figure 4: is there any correlation in panels E and F? I think R^2 should be provided in all panels.

Figure 5: Except for glycoproteins, there's not much of a consistent pattern based on type.

Discussion (275-286): this examination of the role of tissue mass and the plausible related hypothesis is useful.

Discussion (367): "self-reported" race and ethnicity because there is no other kind

Throughout: According to Fraser and other laboratory medicine thought leaders, "reference interval" is technically more accurate than "reference range"

# Author comment: Author response to Decision Letter 3

**DOI:** 10.1371/journal.pbio.3003864.r005

25 May 2026

Attachment Submitted filename: Response to Reviewers R3.docx

# Editor report: Decision Letter 3

**DOI:** 10.1371/journal.pbio.3003864.r006

**Authors:** Ines Alvarez-Garcia, Ines Alvarez-Garcia, Ines Alvarez-Garcia, Ines Alvarez-Garcia

5 Jun 2026

Dear Dr Fuchs,

Thank you for the submission of your revised Research Article entitled "The total mass, copy number, and distribution of hormones in the human bloodstream" for publication in PLOS Biology. On behalf of my colleagues and the Academic Editor, James Ferrell, I am delighted to let you know that we can in principle accept your manuscript for publication, provided you address any remaining formatting and reporting issues. These will be detailed in an email you should receive within 2-3 business days from our colleagues in the journal operations team; no action is required from you until then. Please note that we will not be able to formally accept your manuscript and schedule it for publication until you have completed any requested changes.

Please take a minute to log into Editorial Manager at http://www.editorialmanager.com/pbiology/, click the "Update My Information" link at the top of the page, and update your user information to ensure an efficient production process.

PRESS

We frequently collaborate with press offices. If your institution or institutions have a press office, please notify them about your upcoming paper at this point, to enable them to help maximise its impact. If the press office is planning to promote your findings, we would be grateful if they could coordinate with biologypress@plos.org. If you have previously opted in to the early version process, we ask that you notify us immediately of any press plans so that we may opt out on your behalf.

We also ask that you take this opportunity to read our Embargo Policy regarding the discussion, promotion and media coverage of work that is yet to be published by PLOS. As your manuscript is not yet published, it is bound by the conditions of our Embargo Policy. Please be aware that this policy is in place both to ensure that any press coverage of your article is fully substantiated and to provide a direct link between such coverage and the published work. For full details of our Embargo Policy, please visit http://www.plos.org/about/media-inquiries/embargo-policy/.

Many congratulations and thanks again for choosing PLOS Biology for publication and supporting Open Access publishing. We look forward to publishing your study.

Sincerely,

Ines

--

Ines Alvarez-Garcia, PhD

Senior Editor

PLOS Biology

ialvarez-garcia@plos.org
